# Genome-wide association study of eigenvectors provides genetic insights into selective breeding for tomato metabolites

**DOI:** 10.1186/s12915-022-01327-x

**Published:** 2022-05-24

**Authors:** Junwei Yang, Bin Liang, Yuemei Zhang, Yun Liu, Shengyuan Wang, Qinqin Yang, Xiaolin Geng, Simiao Liu, Yaoyao Wu, Yingfang Zhu, Tao Lin

**Affiliations:** 1 State Key Laborary of Agrobiotechnology, Beijing Key Laboratory of Growth and Developmental Regulation for Protected Vegetable Crops, College of Horticulture, China Agricultural University, Beijing, 100193 China; 2 College of Horticulture, China Agricultural University, Beijing, 100193 China; 3 State Key Laboratory of Plant Genomics, and National Center for Plant Gene Research, Institute of Genetics and Developmental Biology, Innovation Academy for Seed Design, Chinese Academy of Sciences, Beijing, 100101 China; 4 Genome Analysis Laboratory of the Ministry of Agriculture, Agricultural Genomics Institute at Shenzhen, Chinese Academy of Agricultural Sciences, Shenzhen, 518124 Guangdong China; 5 Institute of Plant Stress Biology, State Key Laboratory of Cotton Biology, Department of Biology, Henan University, Kaifeng, 475001 China; 6 Present address: College of Horticulture, China Agricultural University, No.2 Yuanmingyuan West Road, Haidian District, Beijing, 100193 China

**Keywords:** Tomato, EigenGWAS, Metabolites, Selection, *SlCGT*

## Abstract

**Background:**

Long-term domestication and intensive breeding of crop plants aim to establish traits desirable for human needs, and characteristics related to yield, disease resistance, and postharvest storage have traditionally received considerable attention. These processes have led also to negative consequences, as is the case of loss of variants controlling fruit quality, for instance in tomato. Tomato fruit quality is directly associated to metabolite content profiles; however, a full understanding of the genetics affecting metabolite content during tomato domestication and improvement has not been reached due to limitations of the single detection methods previously employed. Here, we aim to reach a broad understanding of changes in metabolite content using a genome-wide association study (GWAS) with eigenvector decomposition (EigenGWAS) on tomato accessions.

**Results:**

An EigenGWAS was performed on 331 tomato accessions using the first eigenvector generated from the genomic data as a “phenotype” to understand the changes in fruit metabolite content during breeding. Two independent gene sets were identified that affected fruit metabolites during domestication and improvement in consumer-preferred tomatoes. Furthermore, 57 candidate genes related to polyphenol and polyamine biosynthesis were discovered, and a major candidate gene *chlorogenate: glucarate caffeoyltransferase* (*SlCGT*) was identified, which affected the quality and diseases resistance of tomato fruit, revealing the domestication mechanism of polyphenols.

**Conclusions:**

We identified gene sets that contributed to consumer liking during domestication and improvement of tomato. Our study reports novel evidence of selective sweeps and key metabolites controlled by multiple genes, increasing our understanding of the mechanisms of metabolites variation during those processes. It also supports a polygenic selection model for the application of tomato breeding.

**Supplementary Information:**

The online version contains supplementary material available at 10.1186/s12915-022-01327-x.

## Background

Plants produce diverse metabolites, which play vital roles in plant growth and development and adaptation to the ever-changing environmental conditions [1]. Besides, they are indispensable bioenergy, nutrition, and medicine resources for human health [2]. Among those detected metabolites, polyphenols are essential metabolites that protect plants against pathogens and herbivores and affect the color and taste of edible organs [3, 4]. Meanwhile, polyamines are differentially regulated in response to various abiotic stresses [5]; they also regulate the accumulation of biomass and fruit quality [6, 7]. Understanding plant metabolites is important for sustainable agriculture and resource conservation. Studies have detected a number of quantitative trait loci (QTLs) for the metabolites in crops, such as tomato [8, 9], rice [10], and maize [11], and making full use of those beneficial loci is invaluable for both phenotyping and diagnostic studies in plants.

Tomato (*Solanum lycopersicum*) has abundant nutrients and biological ingredients for human health and is known as the world’s leading vegetable crop. The global tomato yield was 181 million tons in 2019, with a gross production value of $100 billion (http://www.fao.org/faostat). Although the genome history and fruit mass- and disease resistance-related QTL have been explored in tomato [8, 9, 12], the fruit quality remains largely unknown. In the long-term domestication and breeding, human beings give priority to tomato yield, disease resistance, and postharvest storage, resulting in the loss of superior loci controlling fruit quality, which has caused consumers’ complaints [9, 13, 14]. Combining metabolic profiling with the variome of diverse core tomato accessions makes it possible to decipher the genetic mechanism of the metabolic traits [15]. Understanding variation at the metabolite level facilitates rebuilding metabolites biosynthetic pathways, which in turn will benefit metabolic engineering of desirable compounds and improve tomato quality. The quantitative and qualitative variations in metabolites have made tomato an attractive model for dissecting the metabolic biosynthesis and degradation mechanisms.

Genome-wide association analysis (GWAS) coupled with metabolomic analysis has been successfully performed in rice [10], maize [11], and tomato [9] with many accessions to explore the genetic mechanism of metabolites. However, most of the metabolic traits, such as sucrose, ascorbate, malate, and citrate, are polygenic [16] and likely controlled by a large number of preexisting genetic variants of small effects [17]. Identifying the polygenic selection on metabolites is a complex and challenging process due to multiple loci simultaneously. However, most studies on metabolites have focused on major loci, such as trigonelline and apigenin 5-O-glucoside in rice [10], carotenoids in maize [18], and fruit acids and volatiles in tomato [19] using population genomic analysis, causing the loss of partial small effect genetic variants. Recently, the GWAS of the first eigenvector from the principal component analysis (PCA) (EigenGWAS) is commonly used to identify loci and genomic regions under selection along the gradients of ancestry [20]. Few gene sets or loci related to complex polygenic traits have been identified in avian [21], cattle [22], maize [23], wheat and barley [24], and rice [25] through EigenGWAS. In addition, EigenGWAS can identify novel domestication/improvement sweeps, which are not recognized by nucleotide diversity (𝜋), and therefore regarded as a complementary method for 𝜋 to reduce the omission of selected sweeps.

The present study conducted EigenGWAS on 331 core tomato accessions from a previous report [12] and analyzed the genomic variations in 258 selected metabolites [15]. Meanwhile, the study identified 217 domestication and 280 improvement sweeps. Furthermore, a major candidate gene *chlorogenate: glucarate caffeoyltransferase* (*SlCGT*) was discovered for the polyphenol trait, and the genetic variations in polyphenol during domestication and genome evolution of tomato were revealed. The discovery of 57 genes associated with the polyphenols and the polyamines provides new insights into the polygenic metabolic traits in tomatoes. The study proposes EigenGWAS as an ideal tool as a supplement of 𝜋 for identifying the genes of polygenic traits in crops and crop genomic regions under selection.

## Results

### Metabolite profiling of tomato fruit

**Fig. 1:**
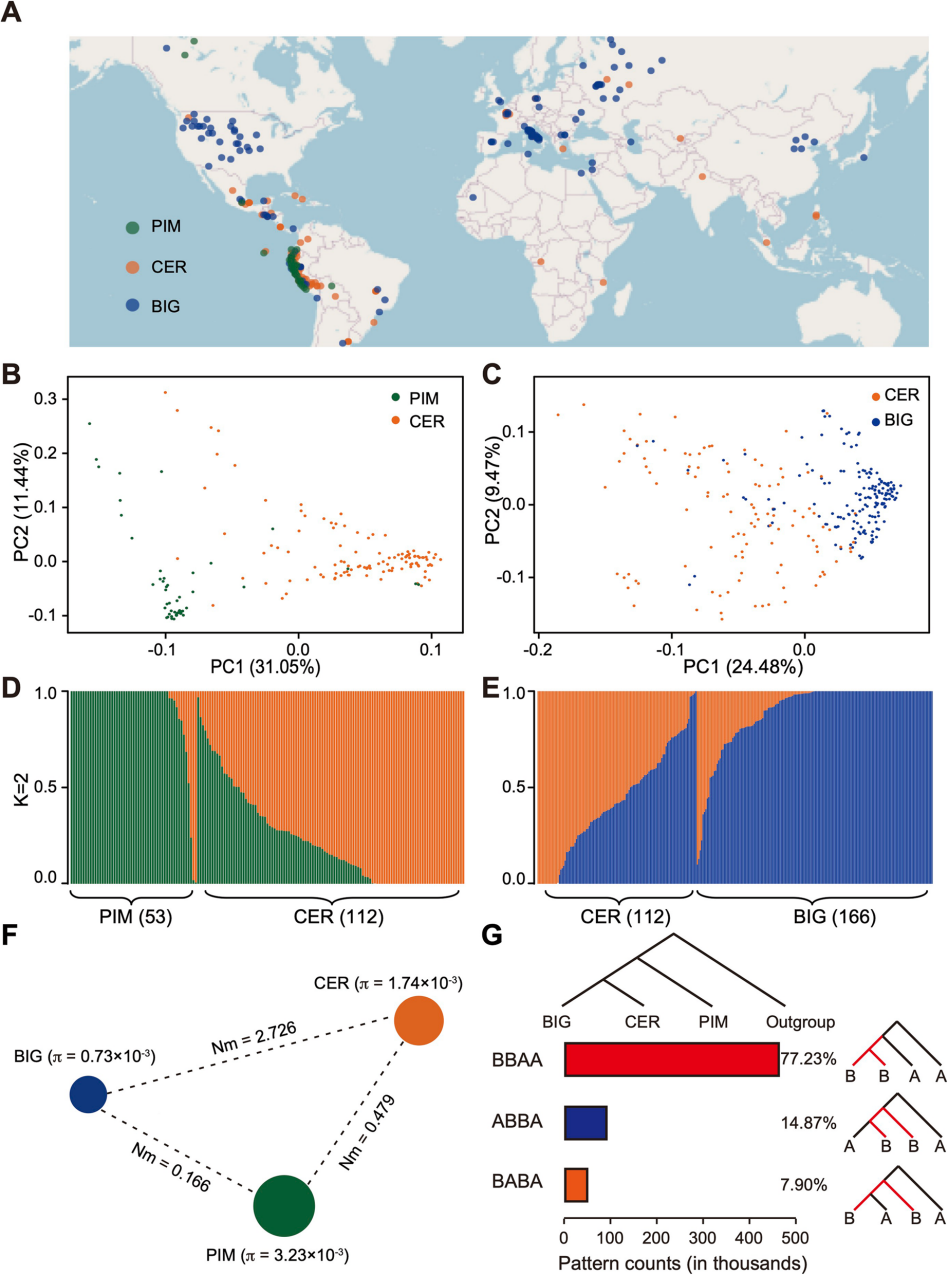
Geographic distribution and population structure of tomato accessions. **A** Geographic distribution of tomato accessions represented by dots on the world map. **B**, **C** Principal component analysis (PCA) of the PIM (*Solanum pimpinellifolium*) and CER (*S. lycopersicum* var. *cerasiforme*) groups (**B**) and the CER and BIG (*S. lycopersicum*) groups (**C**) performed using 136,778 and 51,081 whole-genome SNPs, respectively. **D**, **E** Model-based cluster analysis with two optimal clusters for the PIM and CER groups (**D**) and the CER and BIG groups (**E**). The *x*-axis lists the different accessions, and the *y*-axis quantifies cluster membership. **F** Summary of nucleotide diversity (𝜋) and gene flow level (Nm) across the PIM, CER, and BIG groups. Values in parentheses represent measures of 𝜋 for each group, and values between pairs indicate Nm. **G** The D (ABBA-BABA) and f4-ratio statistics were used to assess evidence of gene flow among the three groups

The study used 331 tomato accessions (Fig. 1A, Additional file 1: Table S1), including 53 *S. pimpinellifolium* (PIM), 112 *S. lycopersicum* var. *cerasiforme* (CER), and 166 *S. lycopersicum* (BIG), from a previous report [12] for metabolite profiling. Among 980 metabolites of these accessions mentioned in an earlier study [15], 258 annotated metabolites, including glycoalkaloids, polyphenols, polyamines, flavonoids, amino acids, phytohormones, vitamins, alkaloids, and terpenoid and their derivatives, were selected through statistical analysis of tomato metabolites content from the PIM, CER and BIG groups (Additional file 1: Table S2). Among these metabolites, 46.34% of glycoalkaloids and 40.63% of polyphenols declined from PIM to CER groups (domestication), and continued to the BIG group (improvement), whereas 51.22% of glycoalkaloids and 31.25% of polyphenols decreased during improvement, after an increase during domestication. In addition, 23.33% of polyamines increased, while 60% decreased during tomato domestication and improvement (Additional file 1: Table S2).

Furthermore, a PCA and model-based cluster analysis based on whole-genome single-nucleotide polymorphisms (SNPs) were conducted for the accessions of PIM and CER, and the accessions of CER and BIG, respectively, to understand the gene flow among the three groups (Fig. 1B–E). The largest principal component (PC1) explained 31.05% of variance related to domestication (Fig. 1B) and 24.48% related to improvement (Fig. 1C), and admixture analysis further verified the existence of genetic structure (Fig. 1D, E). Besides, the gene flow (Nm) analysis revealed a medium Nm between the PIM and CER groups (0.479), a high Nm between the CER and BIG groups (2.726), and a low Nm between the PIM and BIG groups (0.166) (Fig. 1F). The ABBA-BABA statistic involves fitting a simple explicit phylogenetic tree model to verify the existence of gene flow between the different tomato groups (Fig. 1G). These observations indicated a large effective population size and relatively high levels of gene flow between the PIM and CER groups, as well as the CER and BIG groups.

### Novel sweeps reveal tomato metabolites

**Fig. 2:**
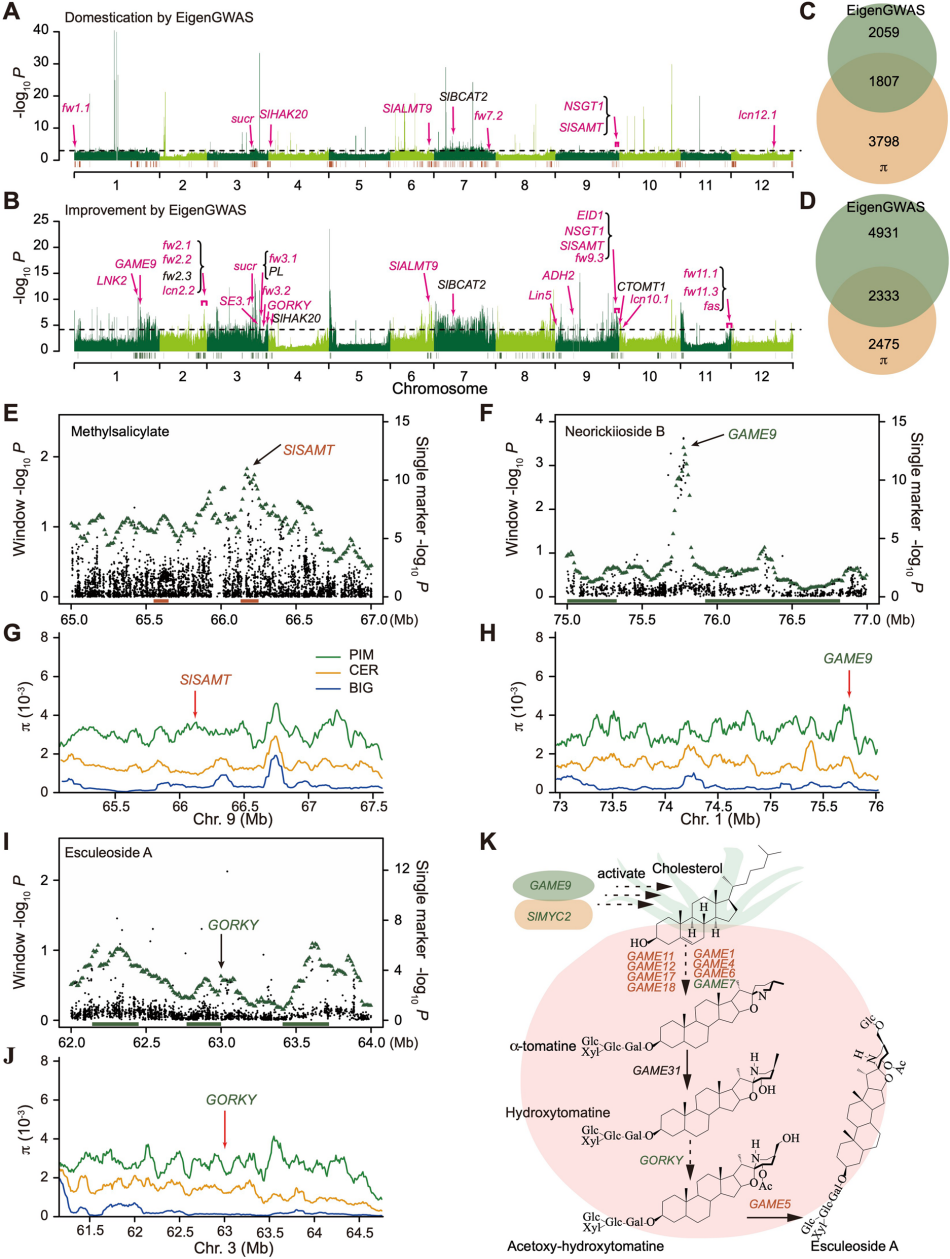
Differentiation and genomic regions under selection among the PIM, CER and BIG groups detected using the EigenGWAS method. **A**–**D** Candidate domestication (217; top 5%, −log_10_ *P* value ≥ 2.98) (**A**) and improvement (280; top 5%, −log_10_ *P* value ≥ 4.19) sweeps (**B**) using EigenGWAS. The orange and green bars above the chromosomes represent the domestication and improvement sweeps identified using the nucleotide diversity (𝜋) method. Candidate genes or quantitative trait loci (QTL) previously reported or identified are marked with different colors. Genes or QTLs marked in red are those detected by the EigenGWAS and 𝜋 methods. Genes or QTLs marked in black are within or surrounding the EigenGWAS peaks. The intersection and union of domestication genes (**C**) and improvement genes (**D**) were identified by EigenGWAS and 𝜋 method. **E**–**J** Local Manhattan plots for single marker GWAS signals (black dots) and 100-kb sliding window GWAS signals (green triangles) of methyl salicylate (**E**), neorickiioside B (**F**), and esculeoside A (**I**). Genomic distribution of 𝜋 of the PIM (green), CER (orange), and BIG (blue) groups for *S-adenosyl-L-methionine: salicylic acid carboxyl methyltransferase* (*SlSAMT*) in chromosome 9 during domestication (**G**), *GLYCOALKALOID METABOLISM 9* (*GAME9*) in chromosome 1 (**H**), and *GORKY* (*Solyc03g120570*) in chromosome 3 (**J**) during improvement. **K** Schematic representation of the core steroidal glycoalkaloid (SGA) metabolic pathway from cholesterol to esculeoside A. Genes in orange and green colors are the domestication and improvement genes, respectively

To identify sweeps during tomato domestication and improvement that were not detected in the previous study [12, 15], EigenGWAS was performed using the PC1 value as a “phenotype.” In total, 217 eigen domestication sweeps (EDS) and 280 eigen improvement sweeps (EIS) were identified and covered 12.98% and 13.97% of the tomato reference genome (version 2.40) (Fig. 2A, B and Additional file 1: Table S3 and Table S4). These EDS and EIS harbored 3866 and 7264 genes, respectively (Fig. 2C, D and Additional file 1: Table S5 and Table S6), in which the number of detected genes was more than those reported by the π method [12]. Then, a gene expression atlas of 399 tomato accessions was constructed using the previously reported transcriptome data obtained at the orange pericarp stage (about 75% ripe) [15] to discover the potential sweep loci related to those selective metabolites. In total, 2572 differentially expressed genes (DEGs) (1219 upregulated and 1353 downregulated) and 1810 DEGs (410 upregulated and 1400 downregulated) were detected during domestication (Additional file 2: Fig. S1A) and improvement (Additional file 2: Fig. S1B), respectively. The GO (Gene Ontology) enrichment analysis showed that the DEGs detected during domestication were involved in response to oxidative stress, transmembrane transport, reproductive process, and regulation of catalytic activity (Additional file 2: Fig. S1C and Additional file 1: Table S7). Meanwhile, the DEGs detected during improvement were involved in chromatin assembly or disassembly, negative regulation of catalytic activity, oxidoreductase activity, and endopeptidase inhibitor activity (Additional file 2: Fig. S1D and Additional file 1: Table S7). Furthermore, the KEGG (Kyoto Encyclopedia of Genes and Genomes) analysis found that the glycolysis/gluconeogenesis, pyruvate metabolism, and phagosome and fatty acid biosynthesis pathways were enriched during domestication (Additional file 2: Fig. S1E and Additional file 1: Table S8), and sesquiterpenoid and triterpenoid biosynthesis, inositol phosphate metabolism, and phenylpropanoid biosynthesis pathways during improvement (Additional file 2: Fig. S1F and Additional file 1: Table S8).

Among the sweep regions, 29 known genes/QTLs related to fruit mass and fruit quality were detected (Fig. 2A, B and Additional file 1: Table S9) [26–48], which was more than that identified by the π method (18 genes/QTLs) [12]. A total of 1807 (Fig. 2C) and 2333 genes (Fig. 2D) detected during domestication and improvement overlapped with the previously identified swept genes using the π method, meanwhile, novel 2059 domestication and 4931 improvement genes were identified through EigenGWAS (Fig. 2C, D). These results indicated those domestication or improvement genes identified solely by the two methods could complement each other. GWAS was performed to validate these sweeps using the important agronomic traits such as methyl salicylate, neorickiioside B, and esculeoside A content and fruit weight (Fig. 2E–J and Additional file 2: Fig. S2). The analysis detected *S-adenosyl-L-methionine: salicylic acid carboxyl methyltransferase* (*SlSAMT*), related to methyl salicylate [41], in EDS183 (120 kb) (Fig. 2E), *GLYCOALKALOID METABOLISM 9* (*GAME9*), regulating steroidal glycoalkaloid [15], in EIS031 (900 kb) (Fig. 2F), *Solyc03g120570* (*GORKY*), preventing tomato bitterness [38], in EIS121 (230 kb) (Fig. 2I), and *Cell Size Regulator* (*CSR/fw11.3*), controlling fruit weight [28], in EIS276 (310 kb) (Additional file 2: Fig. S2A). Furthermore, the 𝜋 intervals of *SlSAMT* (𝜋_PIM_/𝜋_CER_ = 3.55) showed lower nucleotide diversity in the CER group than in the PIM group (Fig. 2G), and those of *GORKY* (𝜋_CER_/𝜋_BIG_ = 9.99), *GAME9* (𝜋_CER_/𝜋_BIG_ = 3.90), and *fw11.3* (𝜋_CER_/𝜋_BIG_ = 8.97) showed lower nucleotide diversity in the BIG group than in the CER group (Fig. 2H, J and Additional file 2: Fig. S2B). These results showed that these cloned genes were indeed selected, which further indicated EigenGWAS was reliable. Neorickiioside B and esculeoside A belong to the steroidal glycoalkaloid (SGA) pathway [15], in which GAME9 activates the SGAs metabolic shift in tomato by co-binding with the SlMYC2 (*Solyc08g076930*) transcription factor, and the bitter α-tomatine is converted to the non-bitter esculeoside A [37, 38]. Among the 13 genes involved in the SGA pathway, eight were located in the domestication sweeps and four within the improvement sweeps (Fig. 2K). Furthermore, *Cell Number Regulator* (*CNR*/*fw2.2*), *cytochrome P450 KLUH* (*SlKLUH/fw3.2*), *WUSCHEL* (*SlWUS*/*lc*), *CLAVATA* (*SlCLV3*/*fas*), *extracellular invertase* (*Lin5*), *NON-SMOKY GLYCOSYLTRANSFERASE1* (*NSGT1*), *sucrose accumulator* (*sucr*), and *Al-ACTIVATED MALATE TRANSPORTER9* (*SlAMT9*) with vital roles in regulating tomato fruit weight [12], locule number [33, 49], and metabolites [9, 40, 42, 47] were also located within the tomato domestication or improvement sweeps. In addition, EigenGWAS identified the novel domestication gene *branched-chain aminotransferases 2* (*SlBCAT2*) [44] in branched-chain amino acid catabolism and the novel improvement genes *catechol-O-methyltransferase* (*CTOMT1*) [43] in guaiacol synthesis, *SlBCAT2* [44], and *pectate lyase* (*PL*) [45] for fruit softening, which were unidentified in the 𝜋 method (Additional file 1: Table S9). These results collectively indicate that EigenGWAS is a powerful tool to detect domestication and improvement signals.

### Identification of selected genes related to polyphenols

Polyphenols are important constituents contributing to fruit quality and an important part of the human diet. Among 258 metabolites, 16 out of 32 polyphenols might have experienced two rounds of human selection (Additional file 1: Table S2). To identify the potential genes related to these polyphenols, GWAS was performed on the PIM and CER, as well as the CER and BIG groups, respectively. In total, 12 significant association signals located within the domestication and improvement sweeps were identified (Additional file 2: Figs. S3 and S4, and Additional file 1: Table S10).

**Fig. 3:**
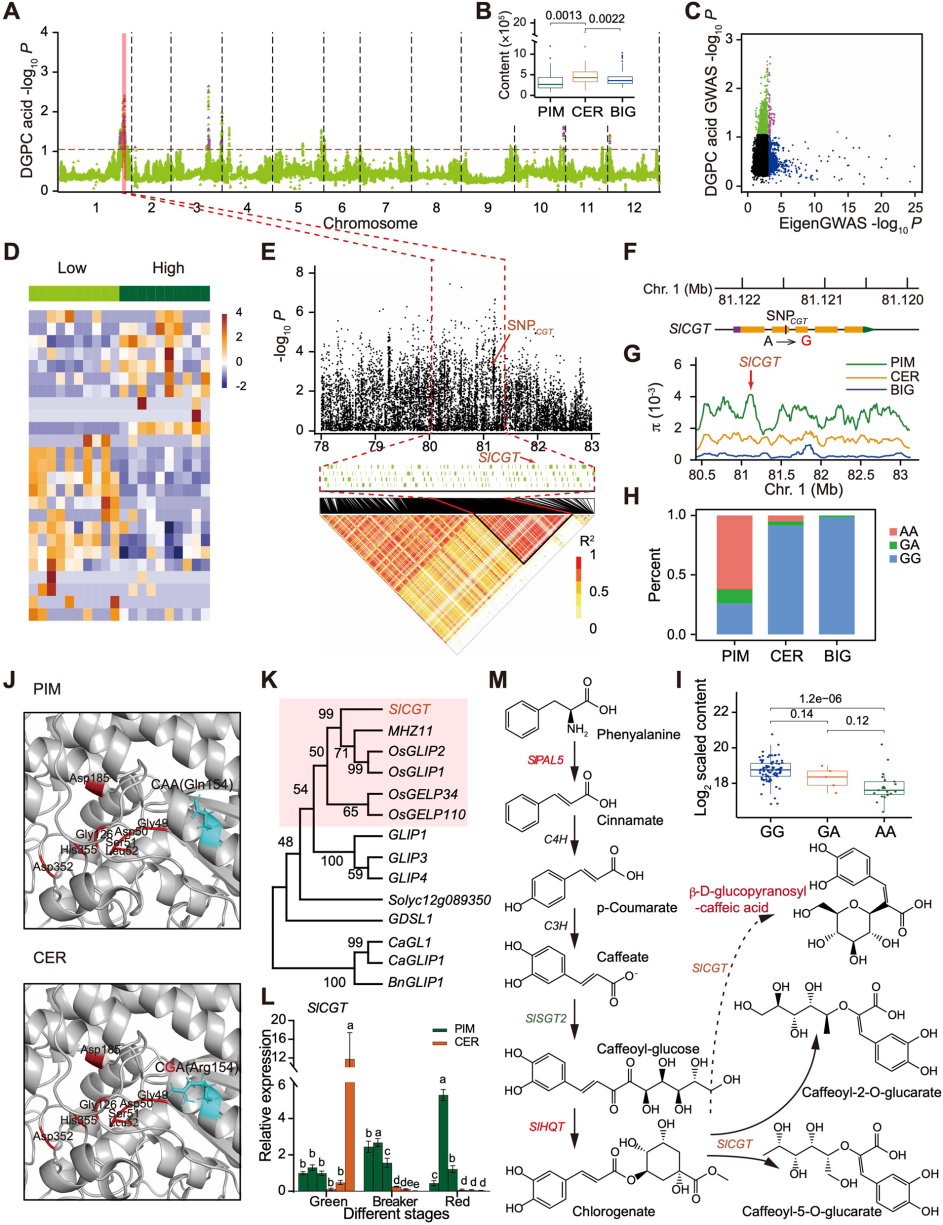
A genomic region for β-D-glucopyranosyl-caffeic acid (DGPC acid) selected under domestication across the PIM and CER groups. **A** Manhattan plot of GWAS on DGPC acid across all chromosomes averaged over 100-kb windows. Color-highlighted regions indicate peaks found in both the GWAS and EigenGWAS analyses. **B** DGPC acid levels in the PIM (green), CER (orange), and BIG (blue) groups are shown. **C** EigenGWAS *P*-values compared with the DGPC acid GWAS *P*-values averaged over 100-kb windows. Green dots indicate windows in the top 1% from GWAS, blue dots indicate windows above the EigenGWAS threshold, and purple dots correspond to the highlighted regions in (**A**). **D** Expression levels of genes in domestication sweep found in both GWAS and EigenGWAS analyses in the low and high DGPC acid tomato accessions. **E** Local Manhattan plot (top), genes in LD block (middle), and a representation of the pairwise R^2^ values (bottom) surrounding the peak on chromosome 1. **F** Gene structure of *chlorogenate: glucarate caffeoyltransferase* (*SlCGT*) and strongly associated SNP_*CGT*_ (−log_10_*P* = 3.24) in the second exon. **G** Genomic distribution of nucleotide diversity (𝜋) of the PIM, CER, and BIG groups within the domestication sweep harboring *SlCGT* on chromosome 1. **H** Distribution of strongly associated SNP_*CGT*_ among the PIM, CER, and BIG groups. **I** Log_2_ transformed DGPC acid content and SNP_*CGT*_ genotype. Comparisons of DGPC acid content in different SNP_*CGT*_ haplotypes among the PIM, CER, and BIG groups are shown. In the box plot, the centerline indicates median; box limits indicate upper and lower quartiles. **J** Protein-structure modeling of SlCGT. Gly49, Asp50, Ser51, and Leu52 are the GDSL motif. The site of Gln154/Arg154 substitution is marked in cyan. **K** Phylogenetic tree generated using SlCGT and its homologs in rice, pepper, *Arabidopsis*, tomato, and rapeseed. **L** Expression profiles of *SlCGT* in fruit pericarp for three low-DGPC acid (PIM) and three high-DGPC acid (CER) tomato accessions, respectively. Data are presented as mean ± SD (*n* = 6, three biological replicates with two technical replicates per accession). **M** Schematic representation of the polyphenol biosynthetic pathway from phenylalanine to DGPC acid in tomato. Genes in orange and green colors represent domestication and improvement genes, respectively

β-D-glucopyranosyl-caffeic acid (DGPC acid) is an important bitter polyphenol that could influence fruit taste. To identify candidate genes related to DGPC acid, GWAS was performed on the PIM and CER groups (Fig. 3A and Additional file 2: Fig. S4A), and the CER and BIG groups (Additional file 2: Fig. S4B and S5A), respectively. The content of this polyphenol increased significantly from the PIM to CER, and then decreased from the CER to BIG group (Fig. 3B), suggesting two rounds of human selection during tomato evolution. In the first round, a strong association signal (*P* = 3.54 × 10^−8^; around 80.04–81.39 Mb) was identified on chromosome 1, which overlapped with EDS051 and EDS052 (0.81 Mb) (Fig. 3A and Additional file 2: Fig. S4A), including 325 genes in the EDS (Fig. 3C). Furthermore, another strong GWAS signal (*P* = 3.13 × 10^−11^; around 79.63–81.79 Mb) was detected in the second round of selection, which overlapped with the improvement region (EIS033, EIS034, and EIS035; 3.24 Mb) (Additional file 2: Figs. S4B and S5A), and 381 genes in the EIS (Additional file 2: Fig. S5B). A comparative genome and transcriptome analysis was performed on these tomato accessions to validate these two signals. During domestication, 25 out of 325 genes were differentially expressed (Fig. 3D and Additional file 1: Table S11), including *SlCGT* (*Solyc01g099020*), encoding a GDSL lipase-like caffeoyltransferase, that resided 0.74 Mb downstream of the strongest association signal in one linkage disequilibrium (LD) block (Fig. 3E). We further analyzed the *SlCGT* sequence and discovered one nonsynonymous site SNP_*CGT*_ in the second exon (Fig. 3F). The π values showed that the *SlCGT* interval was markedly reduced in the CER group compared to the PIM group (Fig. 3G), indicating that *SlCGT* was indeed selected. Haplotype AA was mainly detected in the low-polyphenol PIM group, whereas haplotype GG was seen in the high-polyphenol CER group (Fig. 3H), suggesting that SNP_*CGT*_ may be related to the DGPC acid content (Fig. 3I). Protein modeling with SWISS-MODEL showed that a polymorphism in *SlCGT* resulted in a glutamine-to-arginine substitution in the conserved α-helix domain of SlCGT close to the enzyme active site (Fig. 3J). The eQTL analysis was conducted in the PIM and CER groups (Additional file 1: Table S12), as well as the CER and BIG groups (Additional file 1: Table S13), and it showed that a trans-eQTL signal (Chr01: 78,787,972) close to *SlCGT* was significantly associated with the expression of *SlCGT* (*P* = 5.14 × 10^−10^) in the PIM and CER groups (Additional file 1: Table S12). The orthologs of this gene include *GDSL lipase 1* (*OsGLIP1*) and *GDSL lipase 2* (*OsGLIP2*) (Fig. 3K), which negatively regulated diseases in rice [50], which is similar to the downregulated expression of *SlCGT* in the CER group in the fruit breaker and red stages (Fig. 3L).

Chlorogenate plays an important role in polyphenol biosynthesis, which occurs via the sequential catalysis of an important precursor, phenylalanine, and chlorogenate could synthesize DGPC acid analogs under the action of SlCGT [51]. Three domestication genes, *SlPAL5* (*Solyc09g007910*), *SlHQT* (*Solyc07g005760*), and *SlCGT*, and three improvement genes, *SlPAL5*, *SlSGT2* (*Solyc09g061860*), and *SlHQT*, were identified in these processes (Fig. 3M). During improvement, 19 candidate genes related to DGPC acid were detected, which were involved in histone modification, pectin lyase-like superfamily protein, ATP-dependent DNA helicase, respiratory burst oxidase, and hexosyltransferase (Additional file 2: Fig. S6 and Additional file 1: Table S11). Together, these results indicate that nonsynonymous mutation in *SlCGT* and a *trans*-eQTL may affect its protein structure and relative expression level, then causing the increase of DGPC acid content during domestication. Meanwhile, 19 improvement genes regulating high DGPC acid content for pest and disease resistance were identified, which probably resulted from poor taste of the berries. However, the function of variation in *SlCGT* needs to be verified by more experiments in the future.

### Identification of selected genes related to polyamines

Polyamines play vital roles in regulating plant growth and development and stress tolerance [52]. In this study, 17 polyamines were found during domestication and 26 during improvement (Additional file 1: Table S2). Among these, N′,N″,N‴-trisinapoylspermine (TSPM), a derivate of spermine, was found, which might have experienced two rounds of human selection (Additional file 1: Table S2).

**Fig. 4:**
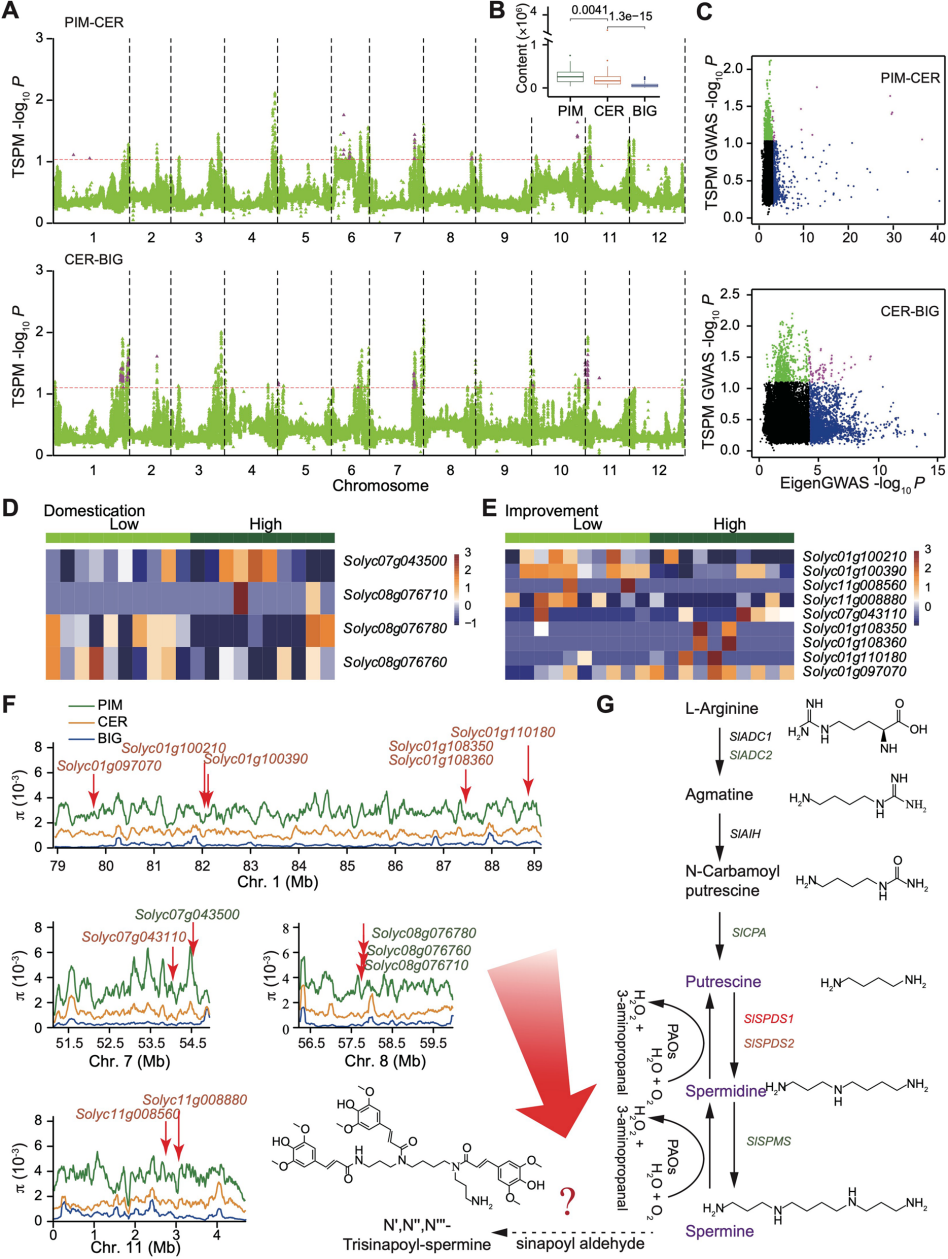
Identification of candidate genes for N',N”,N”'-trisinapoylspermine (TSPM) in tomato. **A** Manhattan plot of GWAS on TSPM across all chromosomes averaged over 100-kb windows for the PIM and CER (Top) and the CER and BIG groups (Bottom). Color-highlighted regions indicate peaks found in both the GWAS and EigenGWAS analyses. **B** TSPM levels in the PIM (green), CER (orange), and BIG (blue) groups are shown. **C** EigenGWAS *P* values compared with the TSPM GWAS *P* values averaged over 100-kb windows for the PIM and CER and the CER and BIG groups. Green dots indicate windows in the top 1% from GWAS, blue dots indicate windows above the EigenGWAS threshold, and purple dots correspond to the highlighted regions in (**A**). **D**, **E** Expression levels of candidate genes in domestication sweep (**D**) and improvement sweep (**E**) found in both the GWAS and EigenGWAS analyses in low and high TSPM content tomato accessions. **F** Genomic distribution of nucleotide diversity (𝜋) for these candidate genes related to TSPM among the PIM, CER, and BIG groups. **G** Schematic representation of spermine biosynthetic pathway from L-arginine to spermine, and then likely to TSPM. Genes in orange and green colors represent domestication and improvement genes, respectively

Due to no single SNP significantly associated with the TSPM during domestication and improvement (Additional file 2: Fig. S7), GWAS of TSPM was performed on the PIM and CER groups and the CER and BIG groups using 100-kb sliding windows (Fig. 4A), and we found the content of TSPM sigificantly decreased from the PIM to CER, then to the BIG group (Fig. 4B). A total of eight and nine association regions, harboring 67 and 353 genes, were further identified during domestication and improvement, respectively (Fig. 4A, C). Among these, four domestication genes and nine improvement genes were differentially expressed (Fig. 4D, E and Additional file 1: Table S14), and the π values showed that these genes were markedly reduced in the CER or BIG group (Fig. 4F). Functional analysis identified one hexosyltransferase gene (*Solyc01g100210*), one glycosyltransferase gene (*Solyc07g043110*), one B-box zinc finger family gene (*Solyc01g110180*), and one AP2-like ethylene-responsive transcription factor (*Solyc11g008560*) (Additional file 1: Table S14), which suggest that these genes might have sustainably reduced the TSPM content during selective breeding of tomato.

L-Arginine initiates spermine biosynthesis, which is catalyzed through more than five processes [53]. In the tomato spermine biosynthetic pathway, five genes, including *SlADC2* (*Solyc01g110440*), *SlCPA* (*Solyc11g068540*), *SlSPDS1* (*Solyc05g005710*), *SlSPMS* (*Solyc08g061970*), and *SlSPDS2* (*Solyc04g026030*), were identified situated in the domestication and improvement sweeps using EigenGWAS or π method (Fig. 4G). In addition, the nonparametric test of Spearman’s rank correlation coefficient showed a higher negative correlation between TSPM and fruit weight (*R*^2^ = 0.40, *P* < 2.2e−16) (Additional file 2: Fig. S8). These results indicated that along with fruit weight, TSPM had undergone a two-step evolution of human selection.

## Discussion

Artificial selection during crop domestication and improvement, in which wild plants are transformed into valuable crops to meet human demands, plays an important role in the improvement of crop yield, quality, and flavor [9, 12, 15]. So far, humans have domesticated several crop varieties and identified a few key genes/QTLs influencing crop growth and development in rice [54], wheat [55], maize [56], and tomato [12]. Yet the mechanisms of crop metabolite variation during domestication and improvement are poorly understood, partly because metabolites are vulnerable to environmental variation [9]. More than 70% of the reported 980 metabolites [15] selected during domestication or improvement provided an interesting direction to explore the impact of artificial selection on metabolite variation among the different tomato groups. An in-depth understanding of the genetic variation mechanism of crop metabolites during domestication and improvement will provide a theoretical basis for improving the poor quality crops and developing excellent quality crops to face the environmental challenge and sustainably meet human needs.

Several statistical methods have been developed to detect the selection signatures, including long-range haplotype (LRH) [57], the integrated haplotype score (iHS) [58], the cross-population extended haplotype homozygosity (XP-EHH) [59], Tajima’s D [60], and π [61]. LRH, XP-EHH, Tajima’s D, and π are not designed for locating genome-wide genetic variants, while iHS is suitable for detecting selection within a single population [58]. However, it is challenging to identify the effective genes that control the quantitative traits dominated by polygenes with minor effects. The present study demonstrates the potential of EigenGWAS, first proposed in human [20], to detect highly significant outlier regions of the genome likely to be under domestication and improvement selection in tomatoes. EigenGWAS has identified numerous candidate gene sets related to the polygenic phenotypes impacted by minor genetic variations [20, 21, 23, 62]. Several studies have used the π method to determine the selected regions along the genome [12, 15, 63]. However, many selected regions were not detected due to the use of a single method. In this study, EigenGWAS identified many novel selective genes not detected by the π method, demonstrating the effectiveness of EigenGWAS in finding loci and genes under selection.

Some metabolites are easily affected by the environment and extremely difficult to quantify, so they remain the major breeding challenges in crops [10, 11, 15]. Among more than 200,000 metabolites in plants [64], few enhance plants’ adaptability to the biotic and abiotic stresses [1], and few affect consumers’ overall liking and fruit flavor intensity [9, 15]. The long-standing crop breeding mainly focuses on yield, disease resistance, long-term storage, which leads to the deterioration of tomato quality. The purpose of this study is to reduce bitterness, modify acidity and sweetness, and cultivate attractive color tomato fruit loved by consumers through understanding the genetic mechanism of fruit metabolites. Polyphenols and polyamines are two major metabolites that influence response to various environmental stimuli, regulate plant growth and development, and affect fruit taste [51, 52, 65]. In this study, *SlCGT* was identified as the most promising candidate gene related to DGPC acid during domestication, increasing DGPC acid content and enhancing disease resistance, then 19 improvement genes regulating DGPC acid to improve the fruit taste. Recent studies have shown that the homologous genes of *SlCGT* in tomato [66], pepper [67, 68], *Arabidopsis* [69, 70], and rapeseed [71] regulated disease resistance and stress tolerance. The enzyme SlCGT is a unique acyltransferase that catalyzes the transfer of caffeoyl moiety from chlorogenate to glucarate and galactarate, forming caffeoylglucarate and caffeoylgalactarate, respectively [72]. It indicated that the glutamine-to-arginine substitution in SlCGT (Fig. 3J) during domestication might affect the GDSL caffeoyltransferase activity and make full use of the chlorogenate to produce more DGPC acid, resulting in influencing fruit taste and enhancing disease resistance. In addition, Tohge et al. [51] provided evidence that SlCGT catalyzes chlorogenate to form caffeoyl-5-O-glucarate and caffeoyl-2-O-glucarate in the polyphenol biosynthesis pathway, consistent with our results that SlCGT catalyzed chlorogenate to DGPC acid in tomatoes. These results show that DGPC acid was probably selected for tuning fruit taste and tomato resistance.

Studies have demonstrated that several genes, such as *ADC1/2*, *SPDS1/2*, *SPMS*, and *SAMDC1/2*, participated in the polyamine metabolic process to cope with abiotic stress and regulated plant growth in *Arabidopsis thaliana* [53, 65]. In this study, 13 candidate genes impacting TSPM content were identified. Two domestication genes, *Solyc06g024220* and *Solyc06g024340* encoding S-adenosylmethionine synthase, involved in spermine synthesis were identified, which are homologs of *SAMDC1/2* (~360 amino acids in length) in *Arabidopsis* [53]. However, their expression levels were not different between the PIM and CER groups due to the incomplete gene structures. We speculated that these two genes mutated during the domestication, resulting in incomplete protein structure (less than 60 amino acids in length). Furthermore, TSPM was found negatively correlated with fruit weight (Additional file 2: Fig. S8), which is not consistent with the result of El-Tarabily et al. [6], who proved that the polyamine-producing actinobacteria enhance biomass production and seed yield in *Salicornia bigelovii*. Thus, the combination of EigenGWAS and GWAS identified a total of 57 candidate genes related to DGPC acid and TSPM in this study, which provides an alternative strategy to uncover important agronomic traits controlled by polygenes, and enhances our understanding of polygenic traits, improves the design and development of molecular breeding in tomato and various other crops; however, further experimental validation is required.

## Conclusions

In summary, we performed EigenGWAS in tomato and identified some novel selective regions and genes that were not identified before, and discovered 57 candidate genes related to polyphenol and polyamine biosynthesis. The present study proposes EigenGWAS as a method complementary to the π method to enhance our understanding of domestication and improvement mechanistic basic and consequence. Furthermore, an alternative idea is that using EigenGWAS and combining the genomic, transcriptomic, and metabolomic data will provide genetic insights into the genetic control of tomato metabolic traits and give a roadmap for polygenic trait improvement.

## Methods

### Collection of phenotypes

The EigenGWAS was based on 331 tomato accessions collected globally in a previous study [12], including 53 *S. pimpinellifolium* (PIM, the closest wild species), 112 *S. lycopersicum* var. *cerasiforme* (CER, cherry tomato), and 166 *S. lycopersicum* (BIG, large-fruited tomato) (Additional file 1: Table S1). Among the three groups, the PIM group has higher genetic diversity and more private SNPs than the CER and BIG groups [10]. The worldwide distribution of tomatoes was plotted using the R package “leaflet” (https://cran.r-project.org/web/packages/leaflet). Transcriptome analysis based on the RNA-seq data of 399 tomato accessions, including 26 PIM, 114 CER, and 259 BIG, reported in Zhu et al. [15]. For the metabolites, we first screened out 362 annotated metabolites among 980 metabolites of 442 tomato lines in the previous report [15], including 31 PIM, 123 CER, and 288 BIG accessions. Then the significance of these metabolites among the PIM, CER, and BIG were estimated by one-way analysis of variance (ANOVA) and Wilcoxon test. In the final, 258 metabolites were considered for further analysis for a significant *P* value less than 0.05 between PIM and CER or CER and BIG groups (Additional file 1: Table S2). The flavor compound methyl salicylate data from Tieman et al. [9] and fruit weight data from Lin et al. [12] were also analyzed in the current study. The correlation between fruit weight and N′,N″,N‴-trisinapoylspermine (TSPM) content from 725 metabolites was tested using Spearman’s rank correlation coefficient [73].

### Population structure and gene flow pattern analysis

Single-nucleotide polymorphisms (SNP) of 331 tomato accessions, genotyped by whole-genome resequencing technology using the Illumina HiSeq 2000 platform, were downloaded from the previous report [12], which was used for population structure and gene flow analysis. The PIM and CER (165 accessions) and the CER and BIG (278 accessions) genotypes were extracted from the PIM, CER, and BIG populations (331 accessions) using python script. Those SNPs with minor allele frequency (MAF) less than 0.05, missing call frequencies greater than 0.1, and linked SNP (*r*^2^ > 0.2) were excluded. A total of 136,778 SNPs and 51,081 SNPs were screened in the PIM and CER, as well as the CER and BIG groups, respectively. A principal component analysis (PCA) was performed on the pruned SNP set using PLINK (v1.9; https://www.cog-genomics.org/plink/1.9) with the command line: plink1.9 –pca, and an R script was used to display the relationship between individuals in different groups in a two-dimensional space. Population structure analysis was performed on the pruned SNP set using the software package ADMIXTURE (v1.3.0; https://dalexander.github.io/admixture) to determine the group membership of each accession with the number of population expected (K) = 2. The GCTA (Genome-wide Complex Trait Analysis, v1.26.0; https://cnsgenomics.com/software/gcta) software was used to analyze the population differentiation index (F_st_) of each SNP locus in all individuals, and the genome-wide average F_st_ was calculated between the PIM and CER, as well as the CER and BIG groups. Gene flow levels (Nm) were analyzed among the three groups, and the Nm value was determined using the formula Nm = (1−F_st_)/4F_st_, and divided into low (0–0.249), medium (0.250–0.99) and high (≥ 1.0) grades [74]. Furthermore, the direction of gene flow between the different groups was estimated using ABBA-BABA statistic in Dsuite [75] (v0.4; https://github.com/millanek/Dsuite).

### Identification of sweeps

The PIM and CER groups (domestication), and the CER and BIG groups (improvement) were screened for between-group selection signatures. To identify domestication and improvement sweeps, we screened a subset of 2,875,396 SNPs in the PIM and CER groups, and 1,704,029 in the CER and BIG groups respectively (MAF > 5% and missing data < 10%). General linear model (GLM) of TASSEL [76] (Trait Analysis by aSSociation, Evolution and Linkage, v5.0; https://www.maizegenetics.net/tassel) was used to conduct EigenGWAS to the first eigenvector during domestication and improvement, with parameters ./run_pipeline.pl -Xmx60g -fork1 -importGuess input_file1 -fork2 -importGuess input_file2 -combine3 -input1 -input2 -intersect -FixedEffectLMPlugin -endPlugin -export output_file. For the EigenGWAS results, the mean *P* values were calculated with a sliding window approach, averaging the signal from all markers within 100 kb windows with a sliding step size of 10 kb along the genome using python script. All windows in the whole genome were sorted from low to high based on the average *P* value, and the top 5% windows were further merged into a single selected region if the distance of the two adjacent windows was less than 200 kb using python script. These selected regions were considered as domestication and improvement sweeps, and the genes within the selected regions were considered domestication/improvement genes (Additional file 1: Tables S3-S6). Moreover, we compared the sweeps/genes identified by EigenGWAS with those identified through nucleotide diversity (π) [12].

### RNA-seq analysis

Differentially expressed genes (DEGs) were identified based on the RNA-seq data of 399 tomato accessions, and the RNA of fruit pericarp was obtained on the orange stage (~75% ripe) [15]. First, the RNA-seq reads from each tomato accession were aligned to the Heinz 1706 genome (v3.0) using HISAT2 [77] (v2.1.0; https://daehwankimlab.github.io/hisat2). Based on the read alignment data, transcripts were assembled with StringTie [77] (v2.0.3; http://ccb.jhu.edu/software/stringtie). After quantifying the expression level of each gene based on ITAG3.2_gene_models.gff, a large gene abundance matrix was constructed containing 35,768 genes from all tomato accessions. The gene expression levels were quantified as fragments per kilobase of exon per million fragments mapped (FPKM). Genes with FPKM equal to zero in all tomato accessions were excluded from subsequent analysis. Furthermore, the FPKM values of the genes were used to identify the DEGs between the PIM and CER groups, and the CER and BIG groups (unpaired samples) using the samWrapper function from R package “DEGseq” in R software [78]. Then, the FPKM values of the DEGs between the different groups were used to plot a heatmap using the R package “pheatmap” (https://cran.r-project.org/web/packages/pheatmap).

### Enrichment analysis

Furthermore, the DEGs between the PIM and CER groups and the CER and BIG groups were used for GO analysis using the R package “TopGO” (http://www.bioconductor.org/packages/release/bioc/html/topGO.html) and KEGG enrichment analysis using the R package “clusterProfiler” [79] (http://www.bioconductor.org/packages/release/bioc/html/clusterProfiler.html).

### Genome-wide association analysis

Furthermore, GWAS was carried out using only those SNPs with MAF > 5% and a missing rate < 10%. A total of 2,875,396 SNPs in the PIM and CER groups and 1,704,029 in the CER and BIG groups were filtered for further analysis. The EMMAX software [80] (Efficient Mixed-Model Association eXpedited vbeta; https://genome.sph.umich.edu/wiki/EMMAX) was used to conduct GWAS. The BN (Balding-Nichols) kinship matrix was constructed based on the filtered SNPs to define the proportion of the randomly selected SNPs for each pair of individuals with default parameters (emmax-kin -v -h -d 10), and the first five principal components were included as fixed effects. The significance level of 0.05 was employed for single testing, and the effective number of independent SNPs (n is the effective number of SNPs) was calculated using the GEC software (Genetic type I Error Calculator v0.2; http://grass.cgs.hku.hk/gec/register.php). The calculated genome-wide significance threshold values (*P*) were 6.10 × 10^−8^ in the PIM and CER groups (*n* = 820,084) and 1.28 × 10^−7^ in the CER and BIG groups (*n* = 391,060), respectively. Manhattan plot displaying the GWAS results using the R package “qqman” (https://cran.r-project.org/web/packages/qqman/).

### Linkage disequilibrium analysis

The SNP genotypes for the PIM and CER groups and SNP physical map were required to display the pairwise linkage disequilibria between SNPs. The SNPs surrounding peaks in the GWAS of β-D-glucopyranosyl-caffeic acid (DGPC acid) were filtered in PLINK1.9, with --maf 0.05 --geno 0.1, the LD heatmap was constructed using the R package ‘LDheatmap’ (https://cran.r-project.org/web/packages/LDheatmap).

### Genetic architecture of the polyphenol and polyamine

To understand the genetic architecture of polyphenol and polyamine. We first performed GWAS on the polyphenol or polyamine using the dataset of the PIM and CER groups, as well as the CER and BIG groups. Then, 100 kb windows sliding with one step of 10 kb along the genome was used to test for an overlap between the most significant EigenGWAS windows (top5 %) and peak windows in the GWAS on the polyphenol and polyamine (top 1%), we screened those genes within these overlap windows for subsequent analysis. Combined with the RNA-seq, gene function information and the variation of the SNPs on or near the screened gene, the candidate genes related to the polyphenol and polyamine were finally screened.

### Protein structure prediction and comparison

To compare the change of variation of SNP_*CGT*_ on SlCGT protein structure, SWISS-MODEL [81] (https://swissmodel.expasy.org) was used to perform homology modeling of SlCGT with default workflow. First, the mutated and non-mutated SlCGT amino acid sequences in FASTA format were inputed. Then, the SlCGT sequence served as a query to search for evolutionary-related protein structures, after selecting a top-ranked template and building model, protein data bank (PDB) format results were downloaded. Finally, PyMOL (v2.4.1; https://www.pymol.org) was used to display and compare the mutated and non-mutated SlCGT protein structure.

### Expression quantitative trait loci (eQTL) analysis

Expression quantitative trait loci (eQTL) analysis links variations in gene expression level to genotypes. The linear regression model of the Matrix eQTL package was used to detect associations for SNP-gene pairs [82] in the PIM and CER, as well as the CER and BIG groups. The expression of each gene was normalized by log_2_(FPKM+1) transformation. Finally, 17,702 genes (missing rate < 80%) in the PIM and CER groups, and 17,899 genes in the CER and BIG groups were obtained to conduct eQTL analysis. We corrected the results with the first ten genotyping principal components and the individual class as the covariates, and the threshold of eQTL analysis is the same as those of GWAS performed in the PIM and CER, as well as the CER and BIG groups, respectively. If SNPs were located within the corresponding gene or less than 30 kb from the transcriptional start point or the end of the gene, it was classified as *cis*-eQTL, otherwise as *trans*-eQTL [15].

### Quantitive RT-PCR (qRT-PCR) analysis

Total RNA was extracted from fruit pericarp in the green, breaker, and red stages using the Quick RNA Isolation Kit (Huayueyang Biotechnology Company), then reversely transcribed applying the PrimeScript^TM^RT reagent kit with gDNA Eraser (TaKaRa). ABI QuantStudio^TM^ 6 Flex (Applied Biosystems, California, USA) was used to quantify the relative expression of target genes. qRT-PCR was performed using a TB Green® Premix EX Taq^TM^ kit 5 μL of TB Green premix (2X), 1 μL of cDNA template, 0.25 μL of each gene-specific primer, 0.25 μL of ROX reference dy, and 3.25 μL ddH_2_O. The reaction conditions were 40 cycles at 95°C for 5 s, 60°C for 34 s after an initial incubation at 95 °C for 15 s, and a dissociation stage was added to ensure specific amplification. *SlEXP* (*Solyc07g025390*) was used as the internal control for qRT-PCR and calculated by the 2^−ΔΔCT^ method. All primers used in this study are presented in Additional file 1: Table S15. Data were given as means ± standard deviation (SD) of three biological replicates with two technical replicates per accession (*n* = 6). A *P* value less than 0.05 (*P* < 0.05) was considered to be statistically significant.

## Supplementary Information

**Additional file 1: Table S1.** Summary of the sampled collection of tomato. **Table S2.** Information of 258 selected metabolites from 980 metabolites. **Table S3.** Putative EigenGWAS and π domestication sweeps. **Table S4.** Putative EigenGWAS and π improvement sweeps. **Table S5.** Genes within the putative EigenGWAS domestication sweeps. **Table S6.** Genes within the putative EigenGWAS improvement sweeps. **Table S7.** GO enrichment analysis of DEGs. **Table S8.** KEGG enrichment analysis of DEGs. **Table S9.** Summary of 29 genes/QTLs associated with tomato plant and fruit. **Table S10.** Summary of 12 significant association signals related polyphenols during domestication and improvement. **Table S11.** β-D-glucopyranosyl-caffeic acid (DGPC acid) selected genes in Eigen domestication and improvement. **Table S12.** The results of eQTL within the PIM and CER groups. **Table S13.** The results of eQTL within the CER and BIG groups. **Table S14.** N',N”,N”'-Trisinapoylspermine (TSPM) selected genes in Eigen domestication and improvement. **Table S15.** The primers of *SlCGT* used for the qRT-PCR experiment.

**Additional file 2: Fig. S1.** Differentially expressed genes (DEGs) and enrichment analysis. Heat map for DEGs between the PIM and CER groups (**A**), as well as the CER and BIG groups (**B**). The Gene ontology (GO) enrichment analysis for DEGs between the PIM and CER groups (**C**), as well as the CER and BIG groups (**D**). The KEGG pathway enrichment analysis for DEGs between the PIM and CER groups (**E**), as well as the CER and BIG groups (**F**). **Fig. S2.** Local Manhattan plot (**A**) and distribution of nucleotide diversity (𝜋) of the PIM, CER, BIG groups for *fw11.3* in chromosome 11 (**B**). Two-Mb zoom of single marker (-log_10_) *P* value for GWAS and 100-kb sliding windows GWAS on fruit weight, and the green bars above the chromosomes denote the identified improvement sweeps by EigenGWAS. **Fig. S3.** GWAS on SIFM0533 and SIFM1279 during domestication, and SIFM0104, SIFM0123, SIFM0154, SIFM0155, SIFM0166, SIFM0656 and SIFM1279 during improvement. Red arrows indicate those significant association signals located in domestication/improvement sweeps using EigenGWAS or 𝜋. Besides these polyphenols, in Supplementary Fig. 4, SIFM0600 were analyzed during domestication and improvement, respectively. **Fig. S4.** GWAS on DGPC acid. Single marker (-log_10_) *P* value for GWAS on DGPC acid during domestication (**A**) and improvement (**B**), respectively. The horizontal axis shows chromosome of tomato, while the vertical axis indicates -log_10_ transformed observed *P* value. **Fig. S5.** A genetic region under improvement across the CER and BIG groups for DGPC acid. **A** Manhattan plot of GWAS on DGPC acid across all chromosome, averaged over 100-kb windows during improvement. Color-highlighted regions indicate peaks found in both the GWAS and EigenGWAS analyses. **B** EigenGWAS *P* values in relation to DGPC acid GWAS *P* values averaged over 100-kb windows. Green dots indicate those windows in the top 1% from GWAS, blue dots indicate those windows above the threshold of EigenGWAS, and purple dots correspond with the highlighted regions in (**A**). **Fig. S6.** Heatmap for those DEGs in the selected sweeps satisfy the EigenGWAS and GWAS in low and high content of DGPC acid during improvement. **Fig. S7.** GWAS on TSPM. Single marker (-log_10_) *P* value for GWAS on TSPM during domestication (**A**) and improvement (**B**), respectively. The horizontal axis shows chromosome of tomato, while the vertical axis indicates -log_10_ transformed observed *P* values. **Fig. S8.** Spearman's rank correlation coefficient between fruit weight and TSPM. The y axis (TSPM content) and x axis (fruit weight) were log_2_ transformed, respectively. Lines and shaded areas are fitted values and 95% confidence limits from general linear models.

## Data Availability

All data generated or analyzed during this study are included in this published article, its supplementary information files, and publicly available repositories. The raw sequence data reported in this study has been deposited in the NCBI Sequence Read Archive (SRA) under accession SRP045767 (https://www.ncbi.nlm.nih.gov/sra/?term=SRP045767) [12]. The RNA-seq data has been deposited under an NCBI BioProject accession PRJNA396272 (https://www.ncbi.nlm.nih.gov/bioproject/?term=PRJNA396272) [15]. Besides, the study used 258 annotated metabolites, selected from 980 metabolites of 442 tomato lines (https://ars.els-cdn.com/content/image/1-s2.0-S009286741731499X-mmc5.xlsx) reported in Zhu et al. [15]. The custom scripts are available at the website Github: https://github.com/Lintao1987/Scripts, and the supporting data associated with the paper are available in the figshare: 10.6084/m9.figshare.19665495.v2.

## Abbreviations

PIM: The closest wild species
CER: Cherry tomato
BIG: Large-fruited tomato
*SlCGT*: *Chlorogenate: glucarate caffeoyltransferase*
QTLs: Quantitative trait loci
GWAS: Genome-wide association analysis
PCA: Principal component analysis
EigenGWAS: GWAS of the first eigenvector from the principal component analysis
𝜋: Nucleotide diversity
Domestication: From PIM to CER groups
Improvement: From CER to BIG groups
Nm: Gene flow
EDS: Eigen domestication sweeps
EIS: Eigen improvement sweeps
DEGs: Differentially expressed genes
GO: Gene Ontology
KEGG: Kyoto Encyclopedia of Genes and Genomes
DGPC acid: β-D-glucopyranosyl-caffeic acid
TSPM: N′,N″,N‴-trisinapoylspermine
