# Supplementary figures and images for: Genome-wide association study of eigenvectors provides genetic insights into selective breeding for tomato metabolites
Source: BMC Biol. 2022 May 24;20:120. doi: 10.1186/s12915-022-01327-x (PMC9128223; doi:10.1186/s12915-022-01327-x)

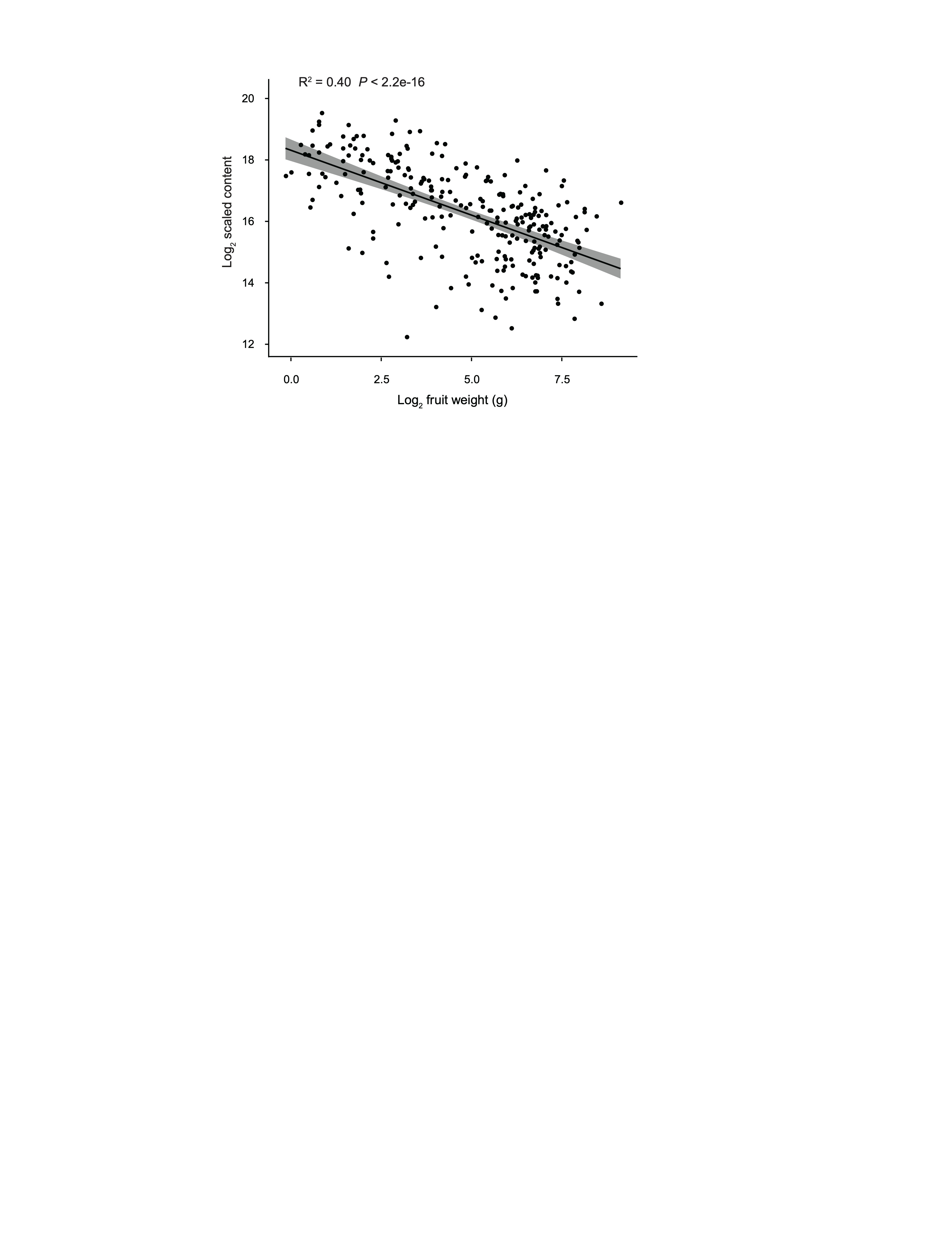

Supplement: Supplementary file 2 — Additional file 2: Fig. S1. Differentially expressed genes (DEGs) and enrichment analysis. Heat map for DEGs between the PIM and CER groups (A), as well as the CER and BIG groups (B). The Gene ontology (GO) enrichment analysis for DEGs between the PIM and CER groups (C), as well as the CER and BIG groups (D). The KEGG pathway enrichment analysis for DEGs between the PIM and CER groups (E), as well as the CER and BIG groups (F). Fig. S2. Local Manhattan plot (A) and distribution of nucleotide diversity (𝜋) of the PIM, CER, BIG groups for fw11.3 in chromosome 11 (B). Two-Mb zoom of single marker (-log10) P value for GWAS and 100-kb sliding windows GWAS on fruit weight, and the green bars above the chromosomes denote the identified improvement sweeps by EigenGWAS. Fig. S3. GWAS on SIFM0533 and SIFM1279 during domestication, and SIFM0104, SIFM0123, SIFM0154, SIFM0155, SIFM0166, SIFM0656 and SIFM1279 during improvement. Red arrows indicate those significant association signals located in domestication/improvement sweeps using EigenGWAS or 𝜋. Besides these polyphenols, in Supplementary Fig. 4, SIFM0600 were analyzed during domestication and improvement, respectively. Fig. S4. GWAS on DGPC acid. Single marker (-log10) P value for GWAS on DGPC acid during domestication (A) and improvement (B), respectively. The horizontal axis shows chromosome of tomato, while the vertical axis indicates -log10 transformed observed P value. Fig. S5. A genetic region under improvement across the CER and BIG groups for DGPC acid. A Manhattan plot of GWAS on DGPC acid across all chromosome, averaged over 100-kb windows during improvement. Color-highlighted regions indicate peaks found in both the GWAS and EigenGWAS analyses. B EigenGWAS P values in relation to DGPC acid GWAS P values averaged over 100-kb windows. Green dots indicate those windows in the top 1% from GWAS, blue dots indicate those windows above the threshold of EigenGWAS, and purple dots correspond with the highlighted regions i [file 12915_2022_1327_MOESM2_ESM.zip › Additional file 2/Figure S8.jpg]

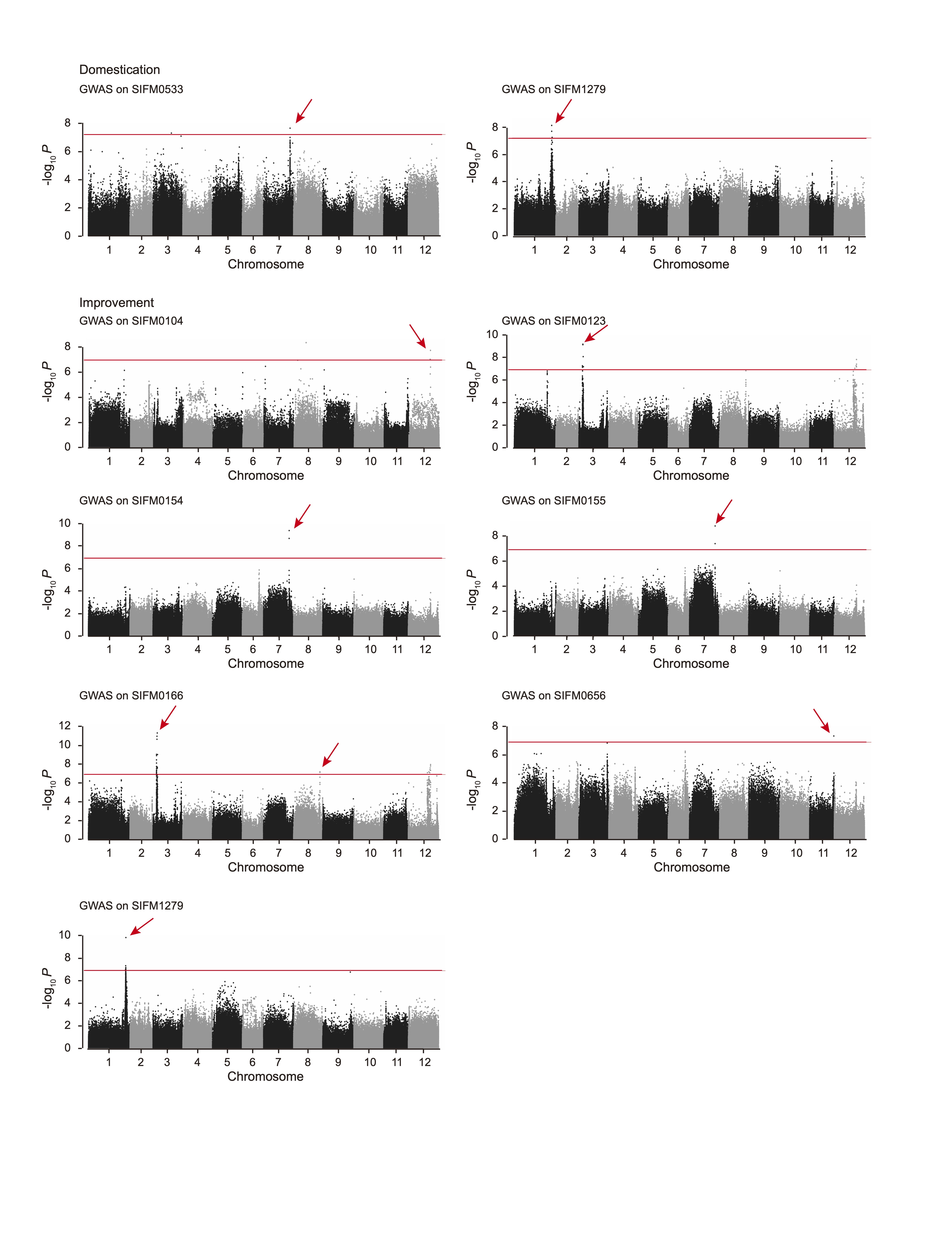

Supplement: Supplementary file 2 — Additional file 2: Fig. S1. Differentially expressed genes (DEGs) and enrichment analysis. Heat map for DEGs between the PIM and CER groups (A), as well as the CER and BIG groups (B). The Gene ontology (GO) enrichment analysis for DEGs between the PIM and CER groups (C), as well as the CER and BIG groups (D). The KEGG pathway enrichment analysis for DEGs between the PIM and CER groups (E), as well as the CER and BIG groups (F). Fig. S2. Local Manhattan plot (A) and distribution of nucleotide diversity (𝜋) of the PIM, CER, BIG groups for fw11.3 in chromosome 11 (B). Two-Mb zoom of single marker (-log10) P value for GWAS and 100-kb sliding windows GWAS on fruit weight, and the green bars above the chromosomes denote the identified improvement sweeps by EigenGWAS. Fig. S3. GWAS on SIFM0533 and SIFM1279 during domestication, and SIFM0104, SIFM0123, SIFM0154, SIFM0155, SIFM0166, SIFM0656 and SIFM1279 during improvement. Red arrows indicate those significant association signals located in domestication/improvement sweeps using EigenGWAS or 𝜋. Besides these polyphenols, in Supplementary Fig. 4, SIFM0600 were analyzed during domestication and improvement, respectively. Fig. S4. GWAS on DGPC acid. Single marker (-log10) P value for GWAS on DGPC acid during domestication (A) and improvement (B), respectively. The horizontal axis shows chromosome of tomato, while the vertical axis indicates -log10 transformed observed P value. Fig. S5. A genetic region under improvement across the CER and BIG groups for DGPC acid. A Manhattan plot of GWAS on DGPC acid across all chromosome, averaged over 100-kb windows during improvement. Color-highlighted regions indicate peaks found in both the GWAS and EigenGWAS analyses. B EigenGWAS P values in relation to DGPC acid GWAS P values averaged over 100-kb windows. Green dots indicate those windows in the top 1% from GWAS, blue dots indicate those windows above the threshold of EigenGWAS, and purple dots correspond with the highlighted regions i [file 12915_2022_1327_MOESM2_ESM.zip › Additional file 2/Figure S3.jpg]

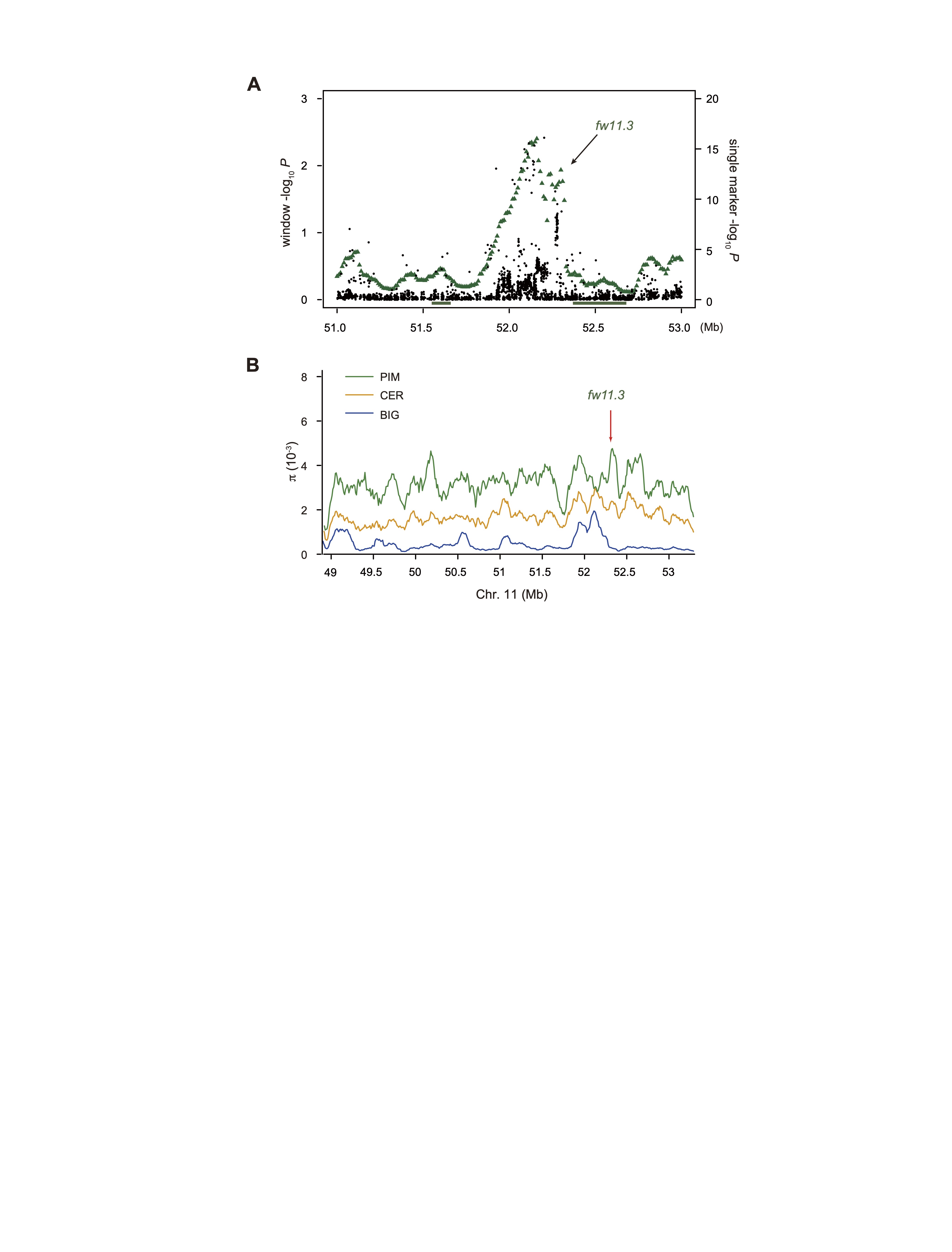

Supplement: Supplementary file 2 — Additional file 2: Fig. S1. Differentially expressed genes (DEGs) and enrichment analysis. Heat map for DEGs between the PIM and CER groups (A), as well as the CER and BIG groups (B). The Gene ontology (GO) enrichment analysis for DEGs between the PIM and CER groups (C), as well as the CER and BIG groups (D). The KEGG pathway enrichment analysis for DEGs between the PIM and CER groups (E), as well as the CER and BIG groups (F). Fig. S2. Local Manhattan plot (A) and distribution of nucleotide diversity (𝜋) of the PIM, CER, BIG groups for fw11.3 in chromosome 11 (B). Two-Mb zoom of single marker (-log10) P value for GWAS and 100-kb sliding windows GWAS on fruit weight, and the green bars above the chromosomes denote the identified improvement sweeps by EigenGWAS. Fig. S3. GWAS on SIFM0533 and SIFM1279 during domestication, and SIFM0104, SIFM0123, SIFM0154, SIFM0155, SIFM0166, SIFM0656 and SIFM1279 during improvement. Red arrows indicate those significant association signals located in domestication/improvement sweeps using EigenGWAS or 𝜋. Besides these polyphenols, in Supplementary Fig. 4, SIFM0600 were analyzed during domestication and improvement, respectively. Fig. S4. GWAS on DGPC acid. Single marker (-log10) P value for GWAS on DGPC acid during domestication (A) and improvement (B), respectively. The horizontal axis shows chromosome of tomato, while the vertical axis indicates -log10 transformed observed P value. Fig. S5. A genetic region under improvement across the CER and BIG groups for DGPC acid. A Manhattan plot of GWAS on DGPC acid across all chromosome, averaged over 100-kb windows during improvement. Color-highlighted regions indicate peaks found in both the GWAS and EigenGWAS analyses. B EigenGWAS P values in relation to DGPC acid GWAS P values averaged over 100-kb windows. Green dots indicate those windows in the top 1% from GWAS, blue dots indicate those windows above the threshold of EigenGWAS, and purple dots correspond with the highlighted regions i [file 12915_2022_1327_MOESM2_ESM.zip › Additional file 2/Figure S2.jpg]

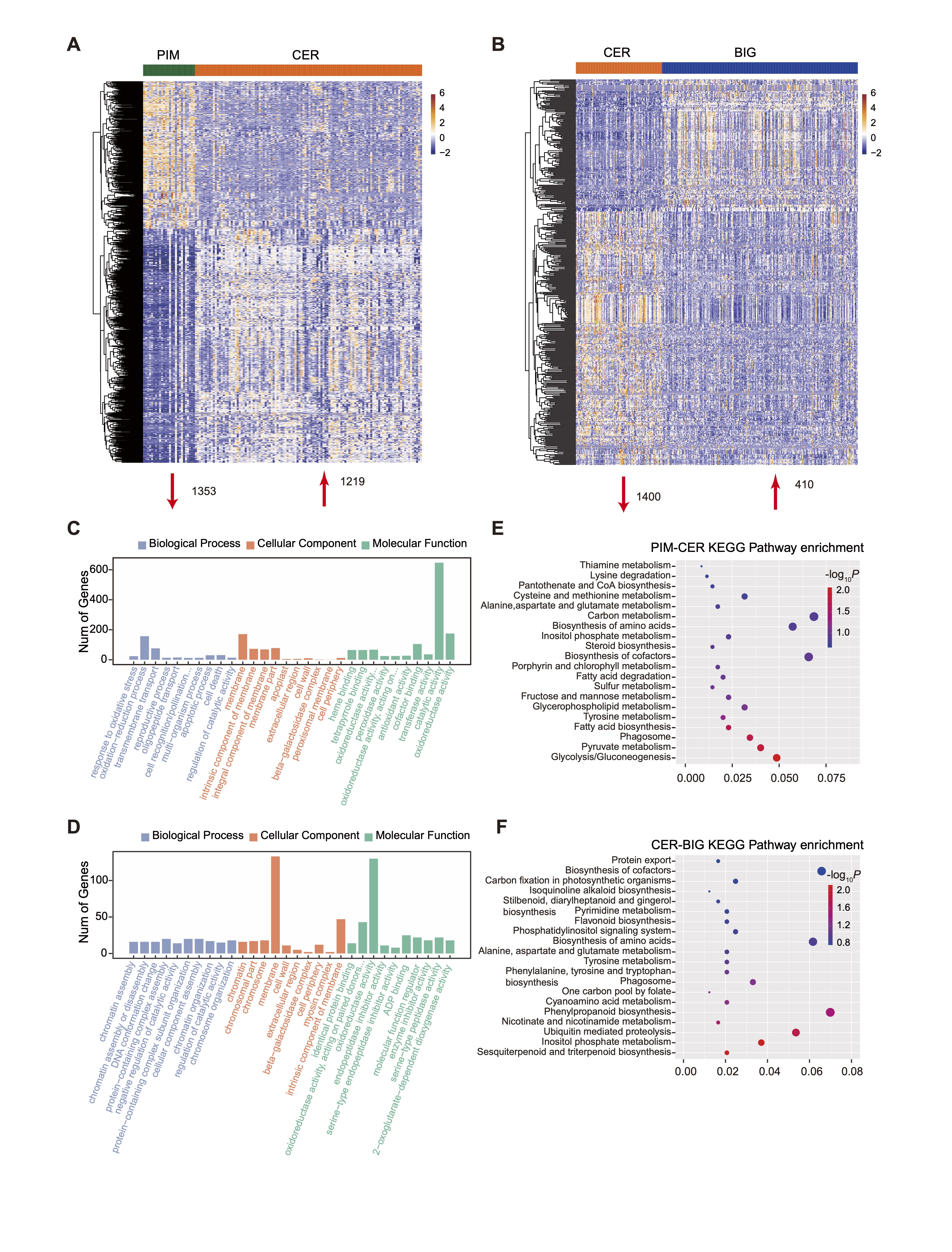

Supplement: Supplementary file 2 — Additional file 2: Fig. S1. Differentially expressed genes (DEGs) and enrichment analysis. Heat map for DEGs between the PIM and CER groups (A), as well as the CER and BIG groups (B). The Gene ontology (GO) enrichment analysis for DEGs between the PIM and CER groups (C), as well as the CER and BIG groups (D). The KEGG pathway enrichment analysis for DEGs between the PIM and CER groups (E), as well as the CER and BIG groups (F). Fig. S2. Local Manhattan plot (A) and distribution of nucleotide diversity (𝜋) of the PIM, CER, BIG groups for fw11.3 in chromosome 11 (B). Two-Mb zoom of single marker (-log10) P value for GWAS and 100-kb sliding windows GWAS on fruit weight, and the green bars above the chromosomes denote the identified improvement sweeps by EigenGWAS. Fig. S3. GWAS on SIFM0533 and SIFM1279 during domestication, and SIFM0104, SIFM0123, SIFM0154, SIFM0155, SIFM0166, SIFM0656 and SIFM1279 during improvement. Red arrows indicate those significant association signals located in domestication/improvement sweeps using EigenGWAS or 𝜋. Besides these polyphenols, in Supplementary Fig. 4, SIFM0600 were analyzed during domestication and improvement, respectively. Fig. S4. GWAS on DGPC acid. Single marker (-log10) P value for GWAS on DGPC acid during domestication (A) and improvement (B), respectively. The horizontal axis shows chromosome of tomato, while the vertical axis indicates -log10 transformed observed P value. Fig. S5. A genetic region under improvement across the CER and BIG groups for DGPC acid. A Manhattan plot of GWAS on DGPC acid across all chromosome, averaged over 100-kb windows during improvement. Color-highlighted regions indicate peaks found in both the GWAS and EigenGWAS analyses. B EigenGWAS P values in relation to DGPC acid GWAS P values averaged over 100-kb windows. Green dots indicate those windows in the top 1% from GWAS, blue dots indicate those windows above the threshold of EigenGWAS, and purple dots correspond with the highlighted regions i [file 12915_2022_1327_MOESM2_ESM.zip › Additional file 2/Figure S1.jpg]

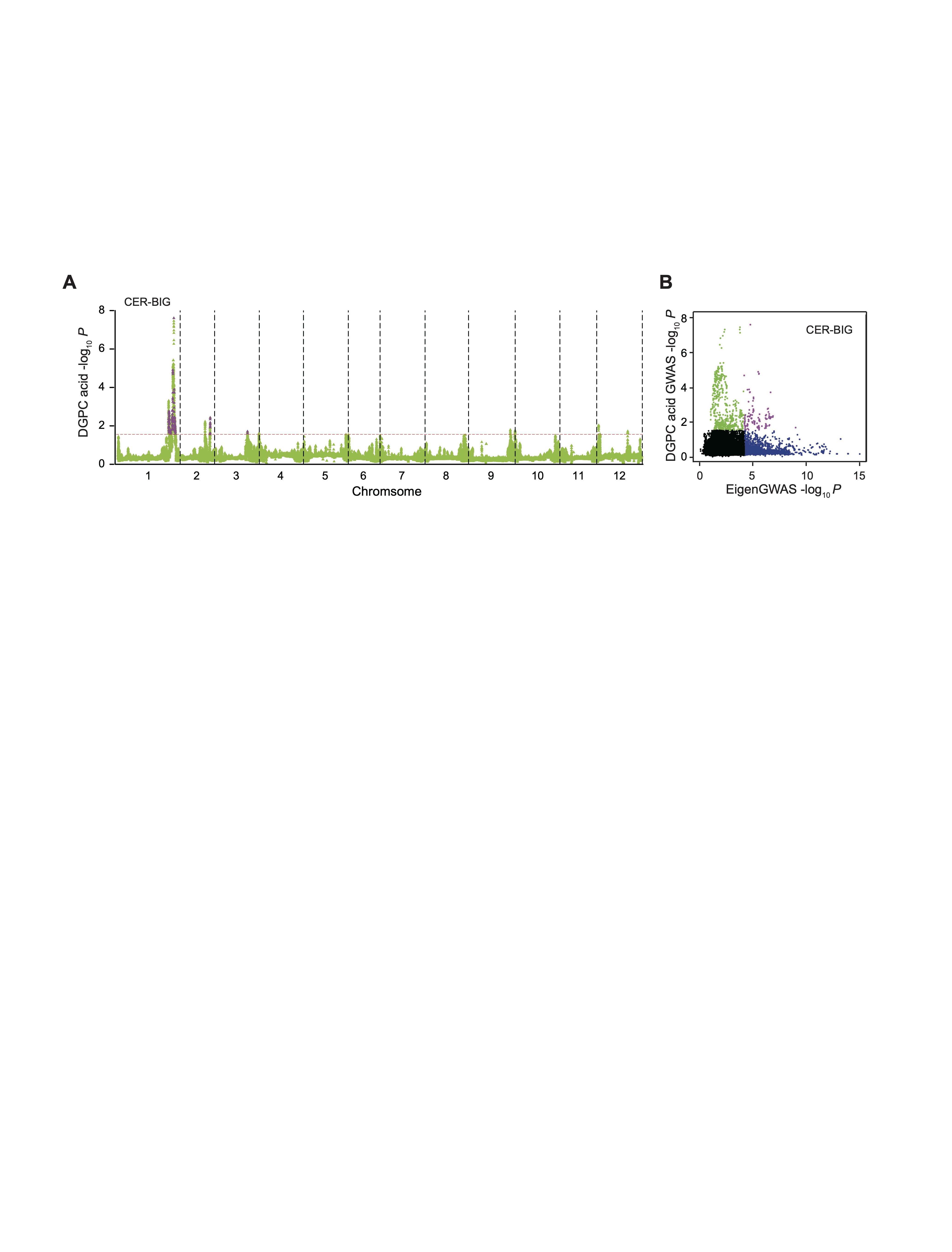

Supplement: Supplementary file 2 — Additional file 2: Fig. S1. Differentially expressed genes (DEGs) and enrichment analysis. Heat map for DEGs between the PIM and CER groups (A), as well as the CER and BIG groups (B). The Gene ontology (GO) enrichment analysis for DEGs between the PIM and CER groups (C), as well as the CER and BIG groups (D). The KEGG pathway enrichment analysis for DEGs between the PIM and CER groups (E), as well as the CER and BIG groups (F). Fig. S2. Local Manhattan plot (A) and distribution of nucleotide diversity (𝜋) of the PIM, CER, BIG groups for fw11.3 in chromosome 11 (B). Two-Mb zoom of single marker (-log10) P value for GWAS and 100-kb sliding windows GWAS on fruit weight, and the green bars above the chromosomes denote the identified improvement sweeps by EigenGWAS. Fig. S3. GWAS on SIFM0533 and SIFM1279 during domestication, and SIFM0104, SIFM0123, SIFM0154, SIFM0155, SIFM0166, SIFM0656 and SIFM1279 during improvement. Red arrows indicate those significant association signals located in domestication/improvement sweeps using EigenGWAS or 𝜋. Besides these polyphenols, in Supplementary Fig. 4, SIFM0600 were analyzed during domestication and improvement, respectively. Fig. S4. GWAS on DGPC acid. Single marker (-log10) P value for GWAS on DGPC acid during domestication (A) and improvement (B), respectively. The horizontal axis shows chromosome of tomato, while the vertical axis indicates -log10 transformed observed P value. Fig. S5. A genetic region under improvement across the CER and BIG groups for DGPC acid. A Manhattan plot of GWAS on DGPC acid across all chromosome, averaged over 100-kb windows during improvement. Color-highlighted regions indicate peaks found in both the GWAS and EigenGWAS analyses. B EigenGWAS P values in relation to DGPC acid GWAS P values averaged over 100-kb windows. Green dots indicate those windows in the top 1% from GWAS, blue dots indicate those windows above the threshold of EigenGWAS, and purple dots correspond with the highlighted regions i [file 12915_2022_1327_MOESM2_ESM.zip › Additional file 2/Figure S5.jpg]

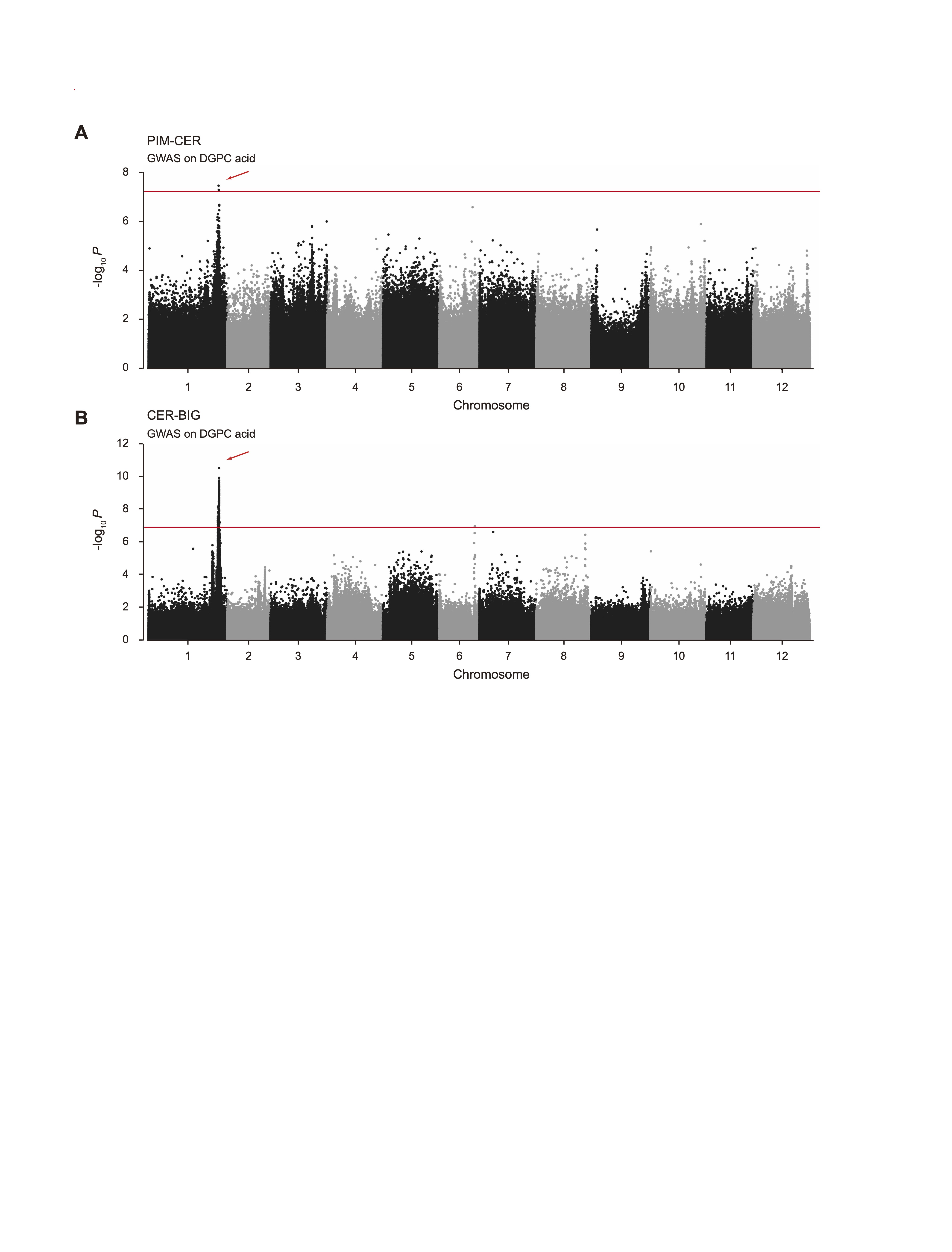

Supplement: Supplementary file 2 — Additional file 2: Fig. S1. Differentially expressed genes (DEGs) and enrichment analysis. Heat map for DEGs between the PIM and CER groups (A), as well as the CER and BIG groups (B). The Gene ontology (GO) enrichment analysis for DEGs between the PIM and CER groups (C), as well as the CER and BIG groups (D). The KEGG pathway enrichment analysis for DEGs between the PIM and CER groups (E), as well as the CER and BIG groups (F). Fig. S2. Local Manhattan plot (A) and distribution of nucleotide diversity (𝜋) of the PIM, CER, BIG groups for fw11.3 in chromosome 11 (B). Two-Mb zoom of single marker (-log10) P value for GWAS and 100-kb sliding windows GWAS on fruit weight, and the green bars above the chromosomes denote the identified improvement sweeps by EigenGWAS. Fig. S3. GWAS on SIFM0533 and SIFM1279 during domestication, and SIFM0104, SIFM0123, SIFM0154, SIFM0155, SIFM0166, SIFM0656 and SIFM1279 during improvement. Red arrows indicate those significant association signals located in domestication/improvement sweeps using EigenGWAS or 𝜋. Besides these polyphenols, in Supplementary Fig. 4, SIFM0600 were analyzed during domestication and improvement, respectively. Fig. S4. GWAS on DGPC acid. Single marker (-log10) P value for GWAS on DGPC acid during domestication (A) and improvement (B), respectively. The horizontal axis shows chromosome of tomato, while the vertical axis indicates -log10 transformed observed P value. Fig. S5. A genetic region under improvement across the CER and BIG groups for DGPC acid. A Manhattan plot of GWAS on DGPC acid across all chromosome, averaged over 100-kb windows during improvement. Color-highlighted regions indicate peaks found in both the GWAS and EigenGWAS analyses. B EigenGWAS P values in relation to DGPC acid GWAS P values averaged over 100-kb windows. Green dots indicate those windows in the top 1% from GWAS, blue dots indicate those windows above the threshold of EigenGWAS, and purple dots correspond with the highlighted regions i [file 12915_2022_1327_MOESM2_ESM.zip › Additional file 2/Figure S4.jpg]

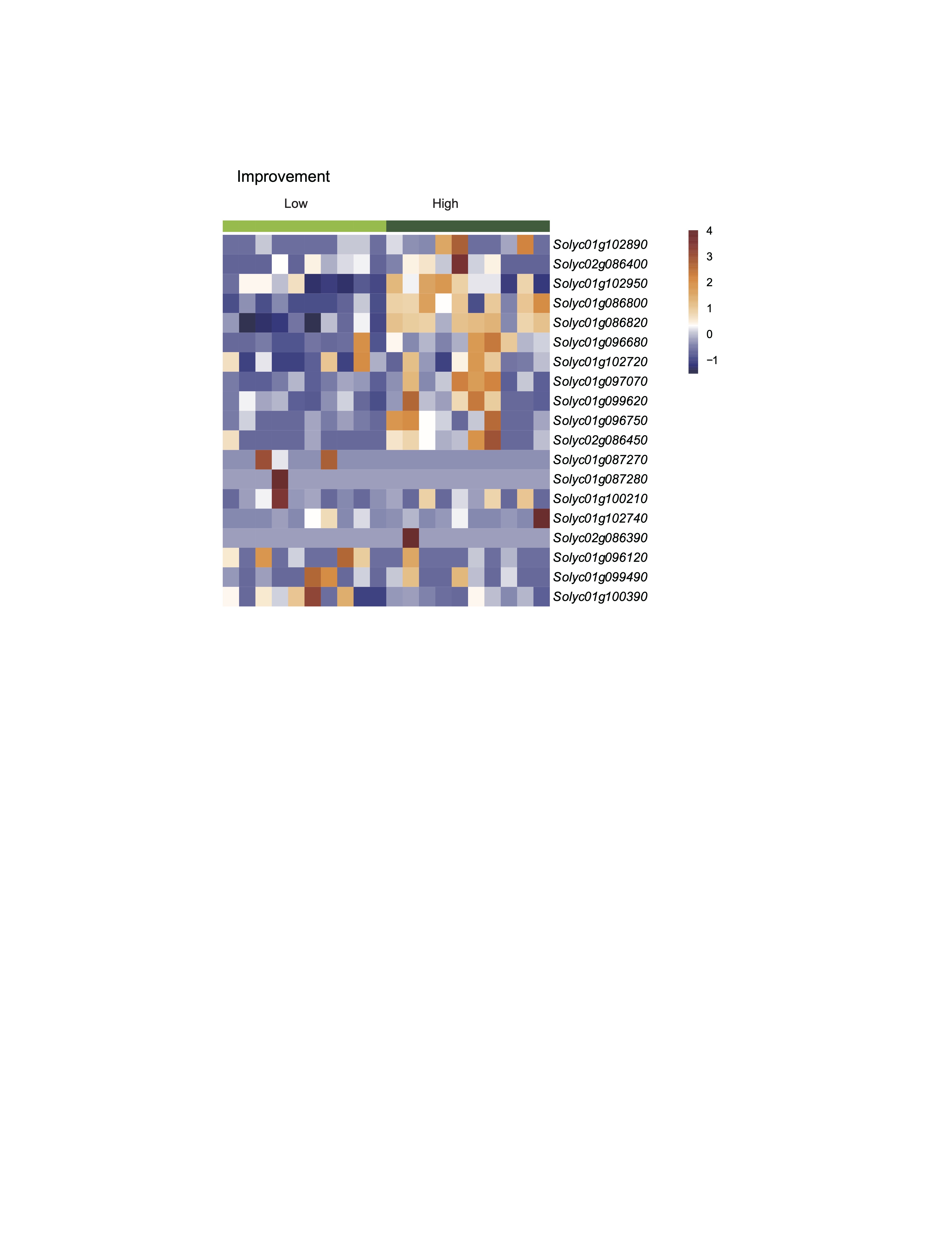

Supplement: Supplementary file 2 — Additional file 2: Fig. S1. Differentially expressed genes (DEGs) and enrichment analysis. Heat map for DEGs between the PIM and CER groups (A), as well as the CER and BIG groups (B). The Gene ontology (GO) enrichment analysis for DEGs between the PIM and CER groups (C), as well as the CER and BIG groups (D). The KEGG pathway enrichment analysis for DEGs between the PIM and CER groups (E), as well as the CER and BIG groups (F). Fig. S2. Local Manhattan plot (A) and distribution of nucleotide diversity (𝜋) of the PIM, CER, BIG groups for fw11.3 in chromosome 11 (B). Two-Mb zoom of single marker (-log10) P value for GWAS and 100-kb sliding windows GWAS on fruit weight, and the green bars above the chromosomes denote the identified improvement sweeps by EigenGWAS. Fig. S3. GWAS on SIFM0533 and SIFM1279 during domestication, and SIFM0104, SIFM0123, SIFM0154, SIFM0155, SIFM0166, SIFM0656 and SIFM1279 during improvement. Red arrows indicate those significant association signals located in domestication/improvement sweeps using EigenGWAS or 𝜋. Besides these polyphenols, in Supplementary Fig. 4, SIFM0600 were analyzed during domestication and improvement, respectively. Fig. S4. GWAS on DGPC acid. Single marker (-log10) P value for GWAS on DGPC acid during domestication (A) and improvement (B), respectively. The horizontal axis shows chromosome of tomato, while the vertical axis indicates -log10 transformed observed P value. Fig. S5. A genetic region under improvement across the CER and BIG groups for DGPC acid. A Manhattan plot of GWAS on DGPC acid across all chromosome, averaged over 100-kb windows during improvement. Color-highlighted regions indicate peaks found in both the GWAS and EigenGWAS analyses. B EigenGWAS P values in relation to DGPC acid GWAS P values averaged over 100-kb windows. Green dots indicate those windows in the top 1% from GWAS, blue dots indicate those windows above the threshold of EigenGWAS, and purple dots correspond with the highlighted regions i [file 12915_2022_1327_MOESM2_ESM.zip › Additional file 2/Figure S6.jpg]

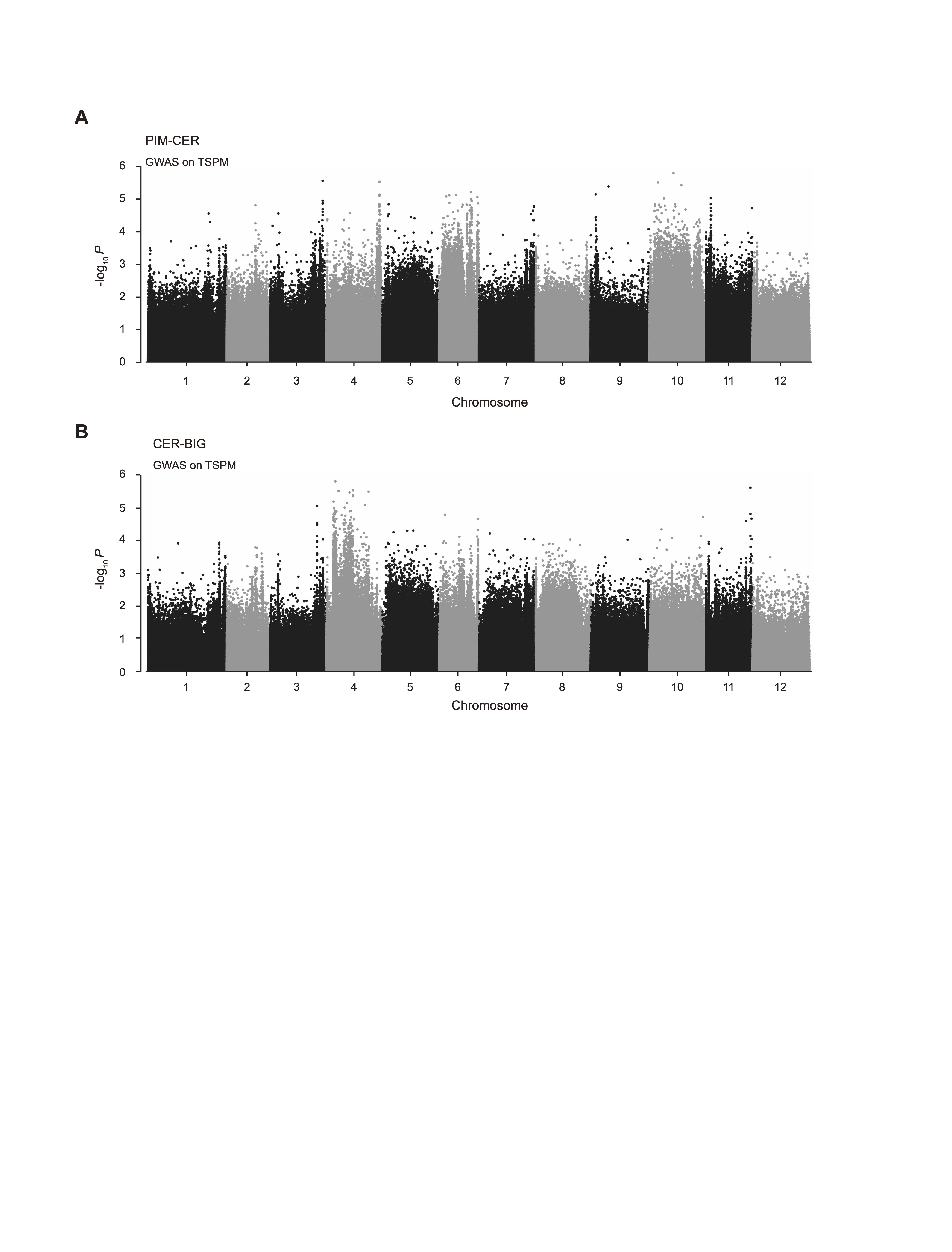

Supplement: Supplementary file 2 — Additional file 2: Fig. S1. Differentially expressed genes (DEGs) and enrichment analysis. Heat map for DEGs between the PIM and CER groups (A), as well as the CER and BIG groups (B). The Gene ontology (GO) enrichment analysis for DEGs between the PIM and CER groups (C), as well as the CER and BIG groups (D). The KEGG pathway enrichment analysis for DEGs between the PIM and CER groups (E), as well as the CER and BIG groups (F). Fig. S2. Local Manhattan plot (A) and distribution of nucleotide diversity (𝜋) of the PIM, CER, BIG groups for fw11.3 in chromosome 11 (B). Two-Mb zoom of single marker (-log10) P value for GWAS and 100-kb sliding windows GWAS on fruit weight, and the green bars above the chromosomes denote the identified improvement sweeps by EigenGWAS. Fig. S3. GWAS on SIFM0533 and SIFM1279 during domestication, and SIFM0104, SIFM0123, SIFM0154, SIFM0155, SIFM0166, SIFM0656 and SIFM1279 during improvement. Red arrows indicate those significant association signals located in domestication/improvement sweeps using EigenGWAS or 𝜋. Besides these polyphenols, in Supplementary Fig. 4, SIFM0600 were analyzed during domestication and improvement, respectively. Fig. S4. GWAS on DGPC acid. Single marker (-log10) P value for GWAS on DGPC acid during domestication (A) and improvement (B), respectively. The horizontal axis shows chromosome of tomato, while the vertical axis indicates -log10 transformed observed P value. Fig. S5. A genetic region under improvement across the CER and BIG groups for DGPC acid. A Manhattan plot of GWAS on DGPC acid across all chromosome, averaged over 100-kb windows during improvement. Color-highlighted regions indicate peaks found in both the GWAS and EigenGWAS analyses. B EigenGWAS P values in relation to DGPC acid GWAS P values averaged over 100-kb windows. Green dots indicate those windows in the top 1% from GWAS, blue dots indicate those windows above the threshold of EigenGWAS, and purple dots correspond with the highlighted regions i [file 12915_2022_1327_MOESM2_ESM.zip › Additional file 2/Figure S7.jpg]
